# High-resolution microbiome analysis of host-rich samples using 2bRAD-M without host depletion

**DOI:** 10.1038/s41522-025-00851-2

**Published:** 2025-11-28

**Authors:** Yuesong Jiang, Jiang Liu, Yufeng Zhang, Lisha Zhou, Enoch Kao, Shuwen Hou, Qianya Niu, Yongxin Liu, Zhenjiang Zech Xu, Tao Ding, Yu-Xiong Su, Yu Liu, Gao Zhang, Xiuping Wang, Fei Teng, Shi Huang

**Affiliations:** 1 Faculty of Dentistry, The University of Hong Kong, Hong Kong SAR, China; 2 Qingdao OE Biotechnology Company Limited, Qingdao, Shandong China; 3 Shenzhen Branch, Guangdong Laboratory of Lingnan Modern Agriculture, Genome Analysis Laboratory of the Ministry of Agriculture and Rural Affairs, Agricultural Genomics Institute at Shenzhen, Chinese Academy of Agricultural Sciences, Shenzhen, China; 4 State Key Laboratory of Food Science and Technology, Nanchang University, Nanchang, China; 5 Department of Immunology and Microbiology, Zhongshan School of Medicine, Sun Yat-Sen University, Guangzhou, China; 6 Qingdao Stomatological Hospital Affiliated to Qingdao University, Qingdao, Shandong China

**Keywords:** Next-generation sequencing, Microbiome, Metagenomics

## Abstract

Characterizing human microbiota in host-dominated samples is crucial for understanding host-microbe interactions, yet is challenged by the high host DNA context (HoC). Current depletion strategies are limited by DNA loss and require immediate processing. In this paper, we introduce 2bRAD-M, a reduced metagenomic sequencing method that enables efficient host-microbe analysis without prior host depletion. Validated on mock samples with >90% human DNA, 2bRAD-M achieved over 93% in AUPR and L2 similarity. In both saliva and oral cancer samples, 2bRAD-M closely matched WMS profiles; in the former, it captured diurnal and host-specific patterns with only 5–10% of the sequencing effort. In an early childhood caries (ECC) study, 2bRAD-M identified key bacterial indicators and distinguished ECC from healthy subjects (AUC = 0.92). By providing high-resolution microbial profiles without host depletion, 2bRAD-M offers a practical and efficient solution for HoC-challenged microbiome research.

## Introduction

Sequencing-based microbiome methodologies have notably expanded our understanding of microbial communities across various human body sites, enabling a progressively nuanced comprehension of host-microbe interactions in health and disease. However, unlike the extensively studied gut microbiota, research on microbial communities in high-host context (HoC) niches, such as saliva and cancer tissues, which typically yield over 90–99% human genome-aligned reads^1,2^, is insufficiently understood, highlighting the urgent need to address the limitations of conventional metagenomic sequencing strategies.

The primary sequencing methods used to establish the taxonomic composition of the microbiome mainly fall into two categories: (i) targeted sequencing of phylogenetic “marker genes” (such as 16S rRNA sequencing) and (ii) whole metagenomic shotgun sequencing (WMS). Although marker-gene-based sequencing is widely employed for characterizing microbial populations in various environmental and host systems, it has several limitations, including (ⅰ) limited taxonomic resolution; (ⅱ) primer bias affecting bacterial clade representation; (ⅲ) PCR bias causing misidentification and inaccurate abundance estimation; and (ⅳ) off-target amplification in HoC samples, where host DNA sequences can be falsely assigned to bacterial taxa^3^. WMS overcomes these hurdles because it analyzes the total DNA content of a sample and doesn’t rely on target-specific primers. However, WMS requires a substantial amount of starting DNA (at least 20–50 ng) and extensive sequencing effort to achieve adequate coverage of microbial genomes in HoC samples^4^. Increasing sequencing depth could improve performance, but is limited by several factors: (i) higher costs from more human reads; (ii) difficulty in detecting low-abundance phenotype-associated microbes in HoC samples; (iii) the prohibitive computational costs of ultra-deep sequencing for many laboratories.

Several strategies have been developed to reduce host DNA in samples, including pre-extraction methods that lyse human cells followed by host DNA degradation, and post-extraction methods that separate microbial DNA based on methylation differences^5^. Pre-extraction techniques, such as lyPMA and MEM, employ a dual-step process that leverages the physical differences between prokaryotic microbial cells and eukaryotic host cells or utilizes selective lysis agents like saponin, complemented by enzymatic or chemical DNA degradation methods (e.g., Benzonase nuclease, propidium monoazide (PMA))^1,6^. These methods, however, confront challenges in maintaining microbial recovery neutrality and preventing DNA loss during processing, which hinders their effectiveness in low-biomass samples^7^. Notably, pre-extraction methods require fresh samples and immediate treatment after collection for effective host depletion. However, they may fail with frozen samples, a more widely accepted and feasible sample type for large-scale clinical microbiome studies^6^. Regarding post-extraction methods, DNA-binding proteins (e.g., methyl-CpG binding protein) and methylation-sensitive endonucleases (e.g., MspJI) are used to enrich microbial sequences and deplete host DNA, respectively^8,9^. The effectiveness of these strategies heavily depends on the methylation status of the target genomes, which may lead to uneven recovery of microbial reads, thus skewing the representation of different microbial lineages^10^; Furthermore, completely excluding host DNA from samples may not be entirely favorable, given that the retention of human DNA yields invaluable insights into the intricate dynamics of microbiome evolution driven by the host factors^11^.

To address host DNA contamination issues, we developed 2bRAD-M^12^—a reduced-representation sequencing method that leverages differences in restriction enzyme site distribution between microbial and human genomes. Microbial genomes encode approximately 150 times more genes than humans^13^, resulting in a higher enzyme site density that preferentially generates microbial-derived 2bRAD-M tags. This selective amplification enhances microbial signal representation in HoC samples (for details, please refer to the sections titled “2bRAD-M sequencing” and “Computational workflow of 2bRAD-M” in the “Methods” section).

While our prior work tested the first version of 2bRAD-M in fecal, skin, and environmental samples, etc.^12^, its performance in prevalent clinical HoC samples—such as fresh saliva and cancer specimens—remained unaddressed, leaving a gap in understanding its utility for high-impact diagnostic applications. Salivary microbiome analysis demonstrates significant clinical value for detecting major oral diseases (e.g., periodontal disease, dental caries^14^) and is linked to systemic conditions, including diabetes, cancers, and neurodegenerative disorder^15–18^. Notably, in oral cancer tissue, microbial signatures can critically influence oral cancer development, response to immunotherapy, and chemotherapy efficacy^19,20^. However, high host DNA levels hide these microbial signals, significantly reducing the detection sensitivity, resolution, and increasing the detection cost. Furthermore, 2bRAD-M’s analytical power critically relies on the quality of the reference database, where the RefSeq database limits its annotation capabilities, i.e., detection and profiling of previously inaccessible microbial “dark matter” in these critical sample types.

In this study, we first employed mock communities to simulate high-host background conditions for assessing the microbial identification and abundance estimation abilities of 2bRAD-M compared to traditional sequencing methods, namely WMS and 16S rRNA sequencing. Notably, the 2bRAD-M Tag database was significantly expanded with GTDB (r202)^21^ and EnsemblFungi^22^ genomes to enhance taxonomic coverage. Subsequently, we evaluated the performance of these three methodologies with real saliva samples and oral cancer specimens, highlighting the high concordance between 2bRAD-M and the current gold-standard method, WMS, and identifying several species whose relative abundance fluctuated diurnally in saliva. Finally, we validated the ability of 2bRAD-M, alongside 16S short-read and long-read sequencing, in distinguishing patients with ECC—a prevalent form of severe dental decay affecting primary teeth in young children—from healthy individuals. Utilizing 2bRAD-M, we identified several key species that significantly enhanced the predictive performance of the ECC model cost-effectively.

## Results

### The practicality of using 2bRAD-M to detect the HoC microbiome through in vitro simulation

**Fig. 1:**
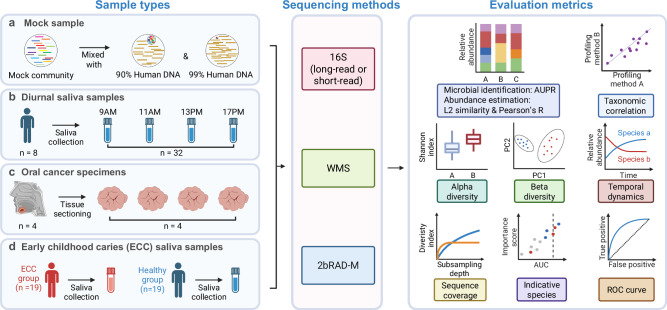
Schematic illustration of the benchmarking workflow in HoC samples. **a** HoC mock communities were created by mixing the standard MSA 1002 (synthetic community) with 90% and 99% human DNA. Sequencing was performed using 2bRAD-M, 16S, and WMS methods. Obtained profiles were evaluated for microbial identification and abundance estimation using AUPR and L2 similarity, respectively. AUPR: Area under the precision-recall curve, a metric that measures the average performance of a classification model in terms of precision and recall across all possible thresholds, particularly suitable for imbalanced datasets; L2 similarity: a metric that measures how close two data points are in space by calculating the reciprocal of the Euclidean distance between them. **b**, **c** For diurnal saliva and oral cancer samples, we employed 2bRAD-M, 16S, and WMS, with WMS results considered as the gold standard. The acquired feature tables underwent L2 similarity analysis for abundance estimation, followed by diversity analysis. Rarefaction analysis was applied to WMS and 2bRAD-M profiles. Furthermore, the temporal dynamics of oscillating species were examined. **d** Early childhood caries (ECC) saliva samples were analyzed using 2bRAD-M, short- and long-read 16S sequencing methods. Diagnostic models of ECC were developed employing Random Forest algorithms on sequencing datasets. The efficacy of these models was evaluated with ROC curves. Indicative species for the 2bRAD-M-derived diagnostic model were visualized on a scatter plot, highlighting their importance scores and corresponding AUC values. This figure was created using BioRender.com.

The performance of 2bRAD-M in microbial identification and abundance estimation was benchmarked against 16S rRNA sequencing and WMS sequencing methods. The conventional 16S method targeting the V4-V5 region was applied to generate genus-level taxonomic profiles, while a recently developed 16S rDNA sequencing protocol, the “5R 16S method”, was employed to predict taxonomic profiles at the species level^23^. Both 16S methods were analyzed using the QIIME2^24^ platform. For WMS sequencing, widely recognized bioinformatic tools, like MetaPhlAn4^25^ and Bracken^26^, were utilized to derive taxonomic profiles down to the genus and species levels (Fig. 1). To critically assess the performance of the profiling tools, the precision-recall curve was used as the primary indicator for microbial identification^27^. It graphically represents precision and recall scores at distinct abundance thresholds. By comparing the generated taxonomic profiles with the ground truth, the area under the precision-recall curve (AUPR) was calculated, providing a single metric for consolidating precision and recall scores and effectively evaluating the presence or absence patterns of microbes^28^. Abundance estimation was assessed based on L2 similarity for a more thorough analysis.

To assess the efficacy of the aforementioned methods in profiling microbial communities amid substantial interference from host DNA, we employed a mock microbial community comprising evenly mixed DNA from 20 bacterial species (across 18 genera)^29^. This composite served as the basis for preparing stocks spiked with 90% and 99% human DNA, respectively. For each type of stock, two technical replicates were generated and subjected to 2bRAD-M, 16S rRNA sequencing, and WMS sequencing methods. Subsequently, the profiles obtained by each method, along with the AUPR and L2 similarity metrics, were then compared to the ground truth across the taxonomic ranks, encompassing the genus and species levels.

2bRAD-M exhibited robust microbial identification and abundance estimation abilities, coupled with commendable technical reproducibility across replicates. In the scenario where host DNA comprises 90% of the sample, the AUPR and L2 similarity scores for 2bRAD-M exhibited exemplary performance, attaining notably high percentages at both the genus and species levels. Conversely, 16S rRNA sequencing yielded lower AUPR and L2 similarity scores across taxonomic levels, whereas WMS demonstrated metric values similar to those of 2bRAD-M. Once the host DNA proportion in the sample reached 99%, the AUPR and L2 similarity scores for 2bRAD-M significantly surpassed those of 16S rRNA sequencing at both the genus and species levels. This highlighted its superior ability in delineating the taxonomic composition of the microbial community, even under HoC conditions. WMS achieved high AUPR but showed reduced L2 similarity, revealing bias in abundance estimation. Notably, significant challenges have been observed in 16S rRNA sequencing, characterized by a pronounced false-positive issue (Fig. 1a, b). The elevated false-positive rate and diminished accuracy in abundance estimation were likely driven by off-target amplification, which exacerbated profile distortion at higher levels of host DNA.

### Benchmarking the taxonomic profiling performance of 2bRAD-M with diurnal saliva samples

**Fig. 2:**
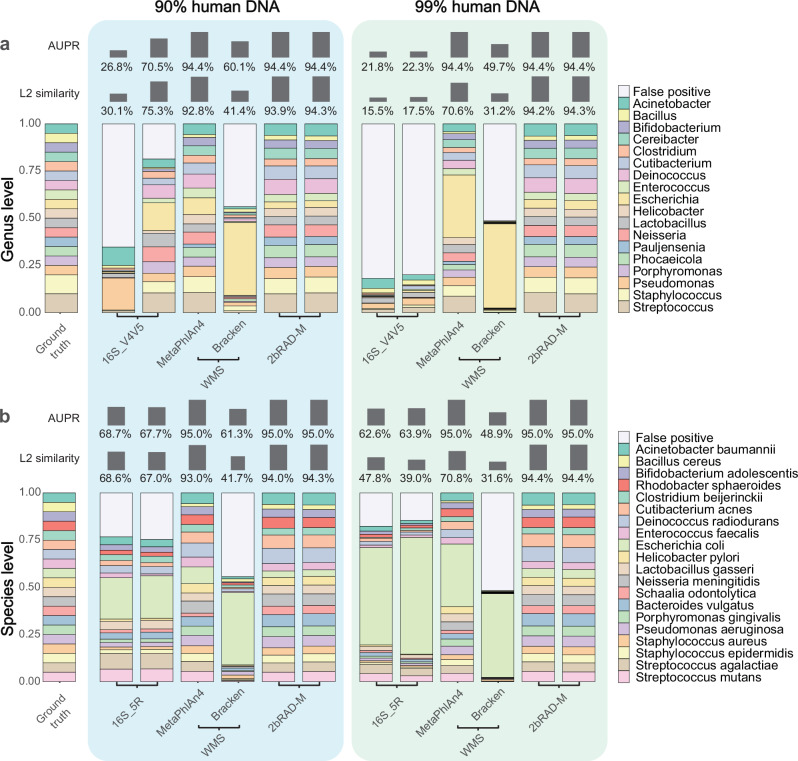
2bRAD-M revealed highly accurate microbiota composition in HoC mock samples. In both (**a**) and (**b**), the left panel shows the taxonomic profiling results of MSA 1002 mixed with 90% human DNA, while the right panel shows results from MSA 1002 mixed with 99% human DNA. In the stacked bar plots, the colored bars indicate bacteria taxa present in the ground truth, while the white bars indicate false positives identified in the profiling results of a given method. AUPR measures the accuracy of microbial identification, and L2 similarity measures the similarity between the ground truth and predicted taxonomic profiles. **a** Genus-level profiling results of the three sequencing methods (16S, 2bRAD-M, WMS) on the same DNA mock community (MSA 1002). **b** Species-level profiling results of the three metagenomics methods.

Elucidating the dynamical changes of the salivary microbiota may reveal mechanisms underlying its association with oral and systemic diseases. To scrutinize the intricate temporal dynamics of salivary microbiota and evaluate the performance of 2bRAD-M on actual HoC samples, a cohort of eight participants was enlisted to provide saliva specimens at four time points: 9 AM, 11 AM, 1 PM, and 5 PM, resulting in a total of 32 samples (referred to as diurnal saliva samples). Each sample was partitioned into three aliquots for analysis using WMS, 2bRAD-M, and 16S rRNA sequencing, respectively (Fig. 2b).

Disparate reference databases across metagenomic profilers may introduce confounding variables when comparing the classification performance of different methods. Therefore, it is imperative to standardize the database before initiating comparative analyses. 16S data analysis depends on using 16S rRNA databases, such as Greengenes and the SILVA database^30^. In contrast, 2bRAD-M relies on GTDB+ EnsemblFungi; WMS data analysis is conducted using MetaPhlAn4, which provides a script to facilitate converting profiles to GTDB-based outcomes. It is noteworthy that the SILVA and GTDB databases do not share a common data universe for microbiome investigations. To enable a more rigorous comparison, we trained a taxonomic classifier utilizing the GTDB within the QIIME2 framework (refer to the code provided in our GitHub Repository). Because real samples lack ground truth, method concordance was assessed using Pearson correlation coefficients (R) and L2 similarity metrics (L2) of taxonomic profiles between WMS and other methods.

**Fig. 3:**
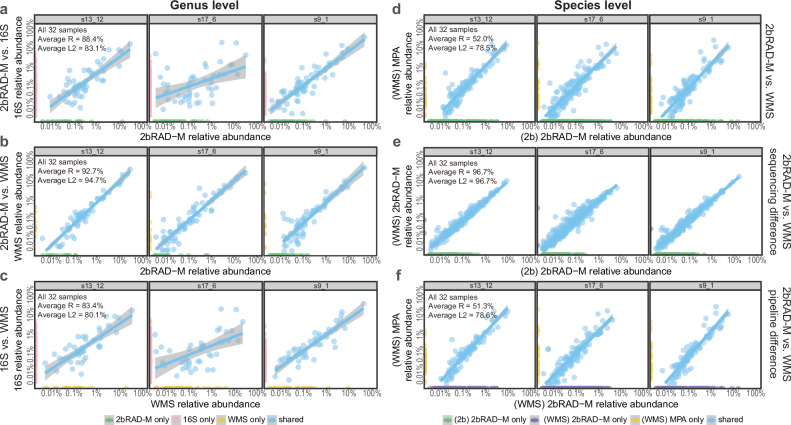
The correlation of microbial abundance at the genus or species level between each pair of three sequencing methods (2bRAD-M, 16S, and WMS) in diurnal saliva samples. Within each group, three out of the total 32 samples were selected to illustrate the correlation between different methodologies. “Average R” and “Average L2” labeled in the figure represent the respective average indices for all 32 samples. The gray box on top of each scatter plot displays the sample ID. Points on the coordinate axis represent unique features identified by the corresponding method, while shared features are indicated by light blue points in the white area of the plot. **a**–**c** Comparative analysis of genus-level taxonomic profiling results obtained from 2bRAD-M, WMS, and 16S rRNA sequencing. The profiles of 2bRAD-M and WMS are highly concordant. **d**–**f** Comparative analysis of species-level taxonomic profiling results of 2bRAD-M and WMS based on overall divergence, sequencing method disparity, and computational pipeline variance. 2bRAD-M and WMS are highly similar in sequencing methods, but significant differences were observed in their corresponding computational pipelines. (WMS) MPA analyzing WMS data using MetaPhlAn4, (WMS) 2bRAD-M analyzing WMS data using the 2bRAD-M computational pipeline, (2b) 2bRAD-M analyzing 2bRAD-M sequencing data with the 2bRAD-M computational pipeline.

After standardization, we compared the taxonomic profiles generated by 2bRAD-M, WMS, and 16S rRNA sequencing at the genus level (Fig. 3a–c). The taxonomic profiles obtained from 2bRAD-M and WMS exhibited high concordance, with an average R of 92.7% and L2 of 94.7% (Fig. 3a; Fig. S1). Conversely, a notable disparity is observed when comparing profiles to those generated using 16S rRNA sequencing: the average R and L2 are 87.4% and 83.9% between 2bRAD-M and 16S, as well as 82.5% and 85.1% between WMS and 16S (Fig. 3b, c; Figs. S2 and S3).

We next scrutinize the effectiveness of 2bRAD-M in species-level taxonomic profiling. Given the inherent limitations in accurately classifying species of 16S data, we chose to exclusively benchmark 2bRAD-M alongside WMS (Fig. 3d–f). Initially, the two approaches were quite dissimilar (average *R* = 52.0% and average L2 = 69.9%) (Fig. 3d; Fig. S4). Although comparing the shared species could enhance the average *R* to 71.1%, the outcome remained suboptimal (Fig. S5). We postulated that the disparity was attributed to variations in both the sequencing techniques and the subsequent computational pipelines. The combination of variances from these two factors might have contributed to the high heterogeneity evident in the overall taxonomic profiles.

To assess the disparity in sequencing techniques, we compared the species-level profiling results from WMS data processed with the 2bRAD-M computational framework, denoted as the (WMS) 2bRAD-M combination, with those from 2bRAD-M sequencing data processed through the 2bRAD-M pipeline, referred to as the (2b) 2bRAD-M combination. Remarkably, the paired results exhibited high degrees of similarity, with an average *R* = 96.7% and L2 = 96.7% (Fig. 3e, Fig. S6). While either data processing combination detected certain unique species, the shared species identified by both combinations represented 98.65% and 99.76% of all reads in the 2bRAD-M data and the WMS data, respectively. This indicates that technique-specific taxa constituted trace biomass fractions. Additionally, correlation analysis conducted on the shared species revealed marginally enhanced metrics in the taxonomic profiling outcomes, with both average R and average L2 96.9% (Fig. S7). Furthermore, the discrepancy between computational pipelines was compared. We juxtaposed the taxonomic profiles derived from WMS data analyzed with MetaPhlAn4, denoted as the (WMS) MPA combination, with those from the (WMS) 2bRAD-M combination, revealing a notable discrepancy (Figs. 3f, S8). These findings demonstrate high concordance between the sequencing techniques of 2bRAD-M and WMS, with disparities in taxonomic profiles primarily caused by distinct computational pipelines.

### 2bRAD-M showed strong agreement with WMS when conducting diversity analyses

**Fig. 4:**
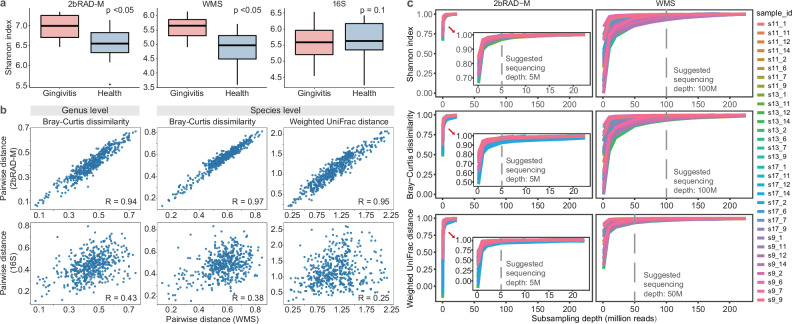
2bRAD-M has a high correlation with whole metagenomic sequencing in diurnal saliva samples. **a** Comparison of alpha diversities (in Shannon index) between health and gingivitis-afflicted samples using 2bRAD-M, WMS, and 16S rRNA sequencing. Kruskal–Wallis test was employed to determine the statistical significance of the observed differences. **b** Correlation between the corresponding distance matrices derived from different methods, with each point denoting the distance between two samples. The distance matrices obtained from 2bRAD-M, whether based on non-phylogenetic distance (Bray–Curtis dissimilarity) or phylogenetic distance (Weighted UniFrac distance), are highly similar to those obtained from WMS. **c** Rarefaction analysis of 2bRAD-M and WMS datasets. In the graph, each colored line represents a sample. From each sample, three subsamples were randomly sampled at several fixed sequencing depths (if the set subsampling volume exceeded the total data volume of the sample, subsampling was stopped). The average alpha and beta diversity indices for each of the three subsamples at each sequencing depth were calculated and compared to the pre-rarefaction results. The suggested sequencing depth for 2bRAD-M is set at the point where adding 1 million reads results in a 1% or less improvement in indices; for WMS, this depth is reached when adding 10 million reads produces no more than 1% similarity gain.”.

To substantiate the capability of 2bRAD-M in yielding biologically equivalent results to other methodologies, particularly WMS, we conducted diversity analyses of these profiles. All saliva samples (*n* = 32) were stratified into the gingivitis group (*n* = 16) and the healthy group (*n* = 16) based on participants’ oral health status, with four individuals diagnosed with gingivitis and four without. Both 2bRAD-M and WMS exhibited significant differences between the two groups in alpha diversity, with Shannon diversities of 7.36 and 12.28, respectively (*p* < 0.05). In contrast, 16S data did not reveal any significant difference between groups (*p* = 0.1) (Fig. 4a). We further performed beta diversity analysis using four distance matrices (Jaccard distance, Bray–Curtis dissimilarity, unweighted UniFrac distance, and weighted UniFrac distance) for the taxonomic profiles generated by each sequencing method. Based on the pseudo-F statistic from the Adonis test, all methods distinguished between gingivitis and healthy individuals. Remarkably, the statistical power of 2bRAD-M closely matched that of WMS and significantly surpassed that of the 16S method (Fig. S9). Both 2bRAD-M and WMS methods demonstrated high effectiveness in discerning microbial diversity variances between gingivitis and health states. Conversely, the 16S method had slightly lower sensitivity to inter-group differences due to its limited resolution.

To evaluate the technical consistency across methods, we performed the Mantel test to assess the similarity between method-specific distance matrices (Fig. 4b). We observed a robust correlation (Pearson’s *R* = 0.97) between the genus-level Bray–Curtis dissimilarity matrices for 2bRAD-M and WMS. However, the similarity between the 16S and WMS matrices was notably lower (*R* = 0.49). At the species level, the Bray–Curtis and weighted UniFrac distance matrices derived from 2bRAD-M and WMS demonstrated strong consistency, with outstanding *R* of 0.97 and 0.95, respectively. The performance of the 16S method was considerably less satisfactory, as indicated by R values of 0.39 and 0.25 for WMS, corresponding to the respective distance matrices. These suggested that 16S yielded less correlated distance matrices compared to WMS and 2bRAD-M. 2bRAD-M and WMS exhibited high concordance in beta diversity analysis in terms of Bray–Curtis and weighted UniFrac distances. While 2bRAD-M and WMS identified unique species, the species identified by both methods were dominant and made up most of the sequencing reads. The identity and relative abundance of shared species exhibited an ultra-high degree of similarity. Collectively, 2bRAD-M technology demonstrates both biological and methodological robustness comparable to WMS when sequencing HoC samples, such as saliva specimens in this study.

### 2bRAD-M can capture similar biological signals to WMS with just 5–10% of the sequencing effort from host-rich samples

The effectiveness of WMS is highly dependent on sequencing depth^31^. To evaluate the minimal sequencing depth for elucidating microbial composition and studying HoC community ecology in 2bRAD-M and WMS sequencing, a series of analyses was performed across a range of increasing sequencing depths. Notably, initial sequencing outputs differed significantly: 32 saliva samples averaged 20 million reads per sample with 2bRAD-M versus 207 million reads with WMS (10 times higher). Both methods showed strong concordance in host DNA quantification. 2bRAD-M demonstrated superior microbial enrichment (mean host reads: 79.56% vs. WMS’s 93.35%; *p* < 0.001), yet yielded biologically consistent host cell proportion estimates (3.28% vs. 3.07%; Table S1). Critically, per-sample human read percentages showed near-perfect inter-method agreement (Pearson’s *R* = 0.93, *p* < 0.001; Fig. S10a), reflecting 2bRAD-M’s enhanced specificity for unbiased microbial DNA enrichment. This systematic reduction in host contamination underscores the method’s dual advantage: (i) minimizing sequencing bias toward host DNA, and (ii) improving cost-efficiency, particularly valuable for large-scale microbiome studies requiring high microbial resolution. Furthermore, the significant correlation in estimated human cell proportions between techniques (Pearson’s *R* = 0.81, *p* < 0.001; Fig. S10b) validates their mutual robustness in distinguishing host-microbial biological signals.

Given 2bRAD-M’s reduced host contamination and lower baseline depth, we next assessed its robustness under depth constraints. We bootstrapped samples down to 0.1, 1, 2, 5, 7.5, 10, 12.5, 15, 17.5, 20, and 22.5 million reads for 2bRAD-M (bootstrap was not conducted if the sequencing depth did not meet the corresponding threshold, totaling 256 samples). WMS data were subsampled to 1, 10, 20, 50, 75, 100, 125, 150, 175, 200, and 225 million sequences per sample (totaling 308 samples). We aimed to determine the sequencing depth necessary for accurate quantification of crucial ecological indicators, including alpha diversity (Shannon index) and beta diversity (Bray–Curtis dissimilarity and weighted UniFrac distance), in both 2bRAD-M and WMS datasets. Notably, at a sequencing depth of 5 million reads per sample, which equals to 609 megabases (Mb) of data, the rarefaction curve of 2bRAD-M reaches saturation and attains Shannon index, Bray–Curtis dissimilarity, and weighted UniFrac distance values that are highly congruent with its deepest read-depth (similarity >99%) (Fig. 4c). This efficiency stems from the ~150-fold higher density of Type IIB restriction sites in microbial vs. host genomes, enabling 2bRAD-M to recover two orders of magnitude more microbial reads per sequencing unit than WMS at equivalent depths (Fig. S11). Consequently, 2bRAD-M needs merely 5–10% of the sequencing data required by WMS to generate the species-resolved taxonomic profile with comparable accuracy. These reductions are especially significant in studies requiring deep sequencing, such as revealing true microbial composition or detecting scarce but ecologically important species in high-host communities, establishing 2bRAD-M as an optimized solution for HoC sample analysis.

### Benchmarking on oral cancer specimens confirms 2bRAD-M’s capability to address HoC challenges

**Fig. 5:**
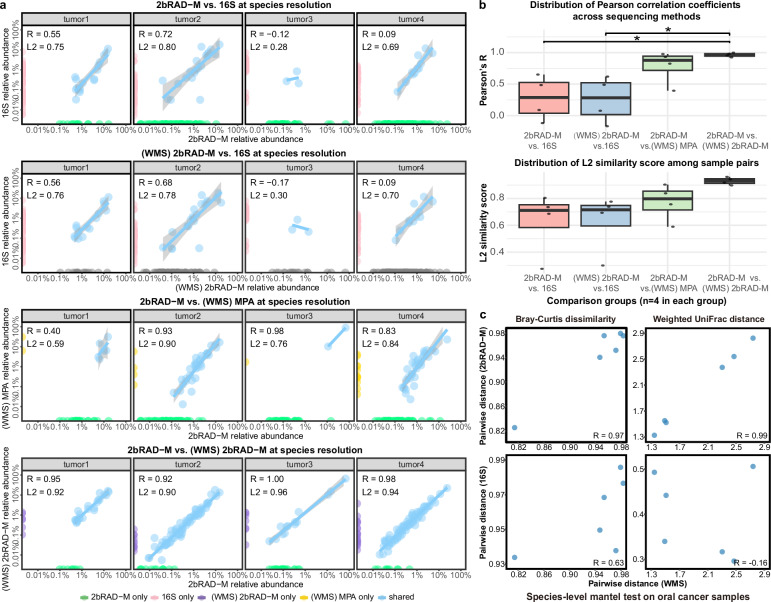
Comparison of species-resolution taxonomic profiles of four tumor samples analyzed by 2bRAD-M, 16S rRNA sequencing, and WMS (processed through both 2bRAD-M and MetaPhlAn4 [MPA] pipelines). WMS results derived from two analytical pipelines (2bRAD-M and Metaphlan4) were denoted as “(WMS) 2bRAD-M” and “(WMS) MPA”, respectively, in the plots. **a** Scatter plots of species-level taxonomic relative abundance (log-scale: 0.01%–100%) for four methodological comparisons: (i) 2bRAD-M vs. 16S, (ii) (WMS) 2bRAD-M vs. 16S, (iii) 2bRAD-M vs. (WMS) MPA, and iv) 2bRAD-M vs. (WMS) 2bRAD-M, with points colored by detection method (2bRAD-M only, 16S only, (WMS) 2bRAD-M only, (WMS) MPA only, or shared taxa). Each plot reports Pearson correlation coefficients (*R*) and L2 similarity scores (L2) per sample. **b** Box plots summarizing distributions of *R* and L2 across the four comparison groups (*n* = 4 samples per group). **c** Species-level Mantel test results based on Bray–Curtis dissimilarity and weighted UniFrac distance matrices. Scatter plots show Mantel correlations (Pearson’s *R*) for each pair of the three methods.

To further demonstrate the generalizability of 2bRAD-M across other HoC sample types, we extended our validation to oral squamous cell carcinoma (OSCC) tissues—a common HoC sample—using four tumor samples from our cohort (IRB approval No.: UW 15-239). Consistent with our analytical approaches for saliva, these tissues underwent 16S rRNA sequencing, 2bRAD sequencing, and WMS, respectively. Similarly, pairwise comparisons (Pearson’s *R* and L2 scores) revealed that 2bRAD-M profiles showed exceptional concordance with WMS-derived profiles (Fig. 5a, b). Critically, when computational pipelines were aligned (2bRAD-M vs. (WMS) 2bRAD-M), species correlations approached near-perfect agreement (*R* and L2 > 96.9%), greatly exceeding correlations between 16S and any other sequencing method (*p* < 0.05, paired *t*-test). Differences in pipelines persisted ((WMS) 2bRAD-M vs. (WMS) MPA), indicating that computational methods, not sequencing strategies, are the main drivers of profiling variations.

Furthermore, we measured the similarity of species-level Bray–Curtis dissimilarity matrices between 16S and WMS profiles or 2bRAD-M and WMS profiles. Strikingly, 2bRAD-M and WMS profiles showed near-perfect concordance (Pearson’s *R* = 0.93), while 16S and WMS comparisons yielded markedly lower agreement (*R* = 0.63, Fig. 5c). Phylogeny-aware UniFrac distance-based analysis further validated this conclusion. 2bRAD-M and WMS profiles still showed near-perfect concordance (*R* = 0.97), while 16S and WMS comparisons yielded no agreement (*R* = −0.16, Fig. 5c). Collectively, these results demonstrate that 2bRAD-M achieves exceptional biological fidelity to gold-standard WMS profiling. Importantly, this high accuracy extends to oral cancer tissues—a challenging host-rich (HoC) sample type—substantially bolstering the method’s clinical applicability beyond saliva-based studies.

### 2bRAD-M captured the subtle changes over time in the saliva microbial communities

**Fig. 6:**
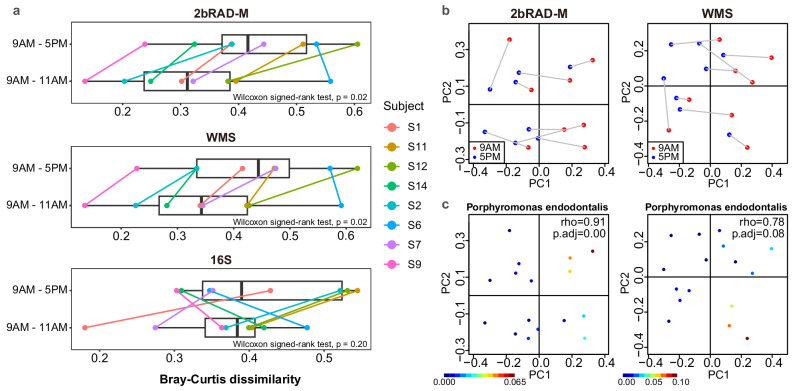
The diurnal pattern of saliva microbiota profiled and compared by three sequencing methods. **a** The box plot shows the Bray–Curtis dissimilarity between each pair of samples collected from the same hosts at different time points (i.e., 9 AM vs. 5 PM, 9 AM vs. 11 AM). Distance values were calculated from the species-level taxonomic profiles produced by 2bRAD-M, WMS and 16S rRNA sequencing. To ensure visual clarity, time point combinations with non-significant differences (e.g., 9 AM vs 1 PM, 11 AM vs 1 PM, 11 AM vs 5 PM and 1 PM vs 5 PM) are not depicted in the plot. The Wilcoxon signed-rank tests were conducted to indicate the significance between the 9 AM–5 PM combination and the 9 AM–11 AM combination. **b** Gradient-like changes in microbial beta diversity over two sampling times, 9 AM (red) and 5 PM (blue). A PCoA plot was used to visualize the distribution of samples collected at the two time points. Gray lines connected samples from the same host. **c** The relative abundance of *Porphyromonas endodontalis* increased along the PC1 axis. Each graph includes a color gradient in the bottom left corner indicating relative abundance, from low (dark blue) to high (brown). Rho represents the Spearman correlation coefficient, and *p.adj* stands for the *p*-value adjusted by Bonferroni correction.

Studies have reported the diurnal oscillation patterns of the oral microbiome^32,33^, but further investigation is needed to elucidate the dynamics of microbial communities at high taxonomic resolution over shorter time frames, such as during the daytime. To benchmark the ability of 2bRAD-M in capturing subtle oscillations within the microbiome of HoC samples, we partitioned the diurnal saliva dataset into four groups based on sampling times (11 AM, 1 PM, and 5 PM compared to their baseline: 9 AM) and computed the Bray–Curtis dissimilarities. Both 2bRAD-M and WMS-based profiles captured the diurnal fluctuations in microbial community structure at the genus level (*p* < 0.01 and *p* = 0.02, respectively), in contrast to 16S rRNA gene sequencing, which showed no significant temporal variation (*p* = 0.10) (Fig. S12). Notably, this temporal pattern was more evident at the species level, where both 2bRAD-M and WMS identified substantial microbial compositional differences between the 9 AM–5 PM and 9 AM–11 AM sampling intervals (Fig. 6a, *p* = 0.02). This observation aligns with previous literature, showing that rhythmic microbial community variations can be detected over the daytime^32^. In contrast, the results from 16S sequencing showed no significant changes in microbial communities at different time points compared to the baseline (*p* = 0.20). These results suggest that 2bRAD-M exhibits high sensitivity in capturing the temporal oscillations in HoC microbial communities.

We further clustered the microbiotas using the principal coordinate analysis (PCoA) plot and identified microbial taxa driving diurnal patterns in the salivary microbiome. In the PCoA plot (Fig. 6b), 5 PM samples consistently localized to the left of their paired 9 AM samples along the PC1 axis, demonstrating unidirectional temporal shifts in community structure (Fig. 6b). Since that PC1 appeared to be the primary descriptor and a good proxy of temporal changes, the relative abundance data of each of key species was projected onto a PCoA plot, visualizing the microbial gradient along PC1 (Fig. 6c). We identified 58 PC1-associated bacterial species using 2bRAD-M and 19 in WMS profiles showing diurnal abundance shifts, with eight species common to both profiles. These shared species-*Filifactor alocis*, *Desulfobulbus oralis*, *Eubacterium M brachy*, *Tannerella forsythia*, *Treponema B denticola*, *Porphyromonas endodontalis*, *F0058 sp000163695*, and *Campylobacter A rectus*-exhibited consistent diurnal depletion from 9:00 AM to 5:00 PM, showing negative correlation with PC1 (Spearman rho > 0.7, *p* < 0.01, Fig. S13). In previous literature, the genus *Porphyromonas* has been shown to exhibit a notable decreasing trend in the saliva of one or multiple individuals in the daytime^32^, consistent with our observations in this study (Fig. 6c). Notably, this trend was confirmed by qPCR measurement of *P. endodontalis* and overall bacteria load in eight samples from four subjects at 9 AM/5 PM time points (selected based on sufficient residual DNA). We demonstrated a strong correlation between qPCR-derived relative abundance and 2bRAD-M measurements (Pearson’s *R* = 0.99, *p* < 0.01; Fig. S14 and Table S2). Therefore, 2bRAD-M provides a promising avenue for studying the subtle dynamics of the microbiome in HoC samples and holds potential applications in assessing the relationship between this variation and the host’s physiological state.

### 2bRAD-M demonstrates a stronger discriminatory power in classifying ECC than amplicon-based metagenomics

Understanding the human microbiome is essential for improving disease classification, treatment, and prevention by leveraging insights from microbiota profiles. The use of oral microbiota to predict disease states, as demonstrated in studies analyzing saliva and plaque samples for diseases like ECC^34^ and gingivitis^35^, is a burgeoning area of research. ECC, affecting nearly half of children worldwide, results in considerable social and economic burdens^36^. It causes permanent dental damage, increasing the risk of further decay and tooth loss over a child’s life. Therefore, timely diagnosis and prevention of ECC are critically important. Recent studies have shown the effectiveness of using species-level taxonomic profiles generated from 16S short-read sequencing of saliva and plaque to predict ECC^34^. However, models based on 16S sequencing data showed limited effectiveness (AUC = 0.68), likely due to the inherent resolution constraints of the technique. Implementing high-resolution sequencing techniques, like 2bRAD-M, could enhance model performance by providing species-resolved and more accurate taxonomic profiles.

To evaluate the performance of 2bRAD-M against conventional sequencing methods in diagnosing ECC, we initiated our study by selecting 19 high-quality saliva samples from each of the ECC and healthy control groups in the study above. These selections were based on the integrity and completeness of data from previous 16S short-read sequencing efforts, which specifically targeted the V3V4 regions. Subsequently, these samples were analyzed using both 2bRAD-M and 16S long-read sequencing. To ensure a consistent and fair comparison across all three methods, we used the GTDB r202 as our reference database.

**Fig. 7:**
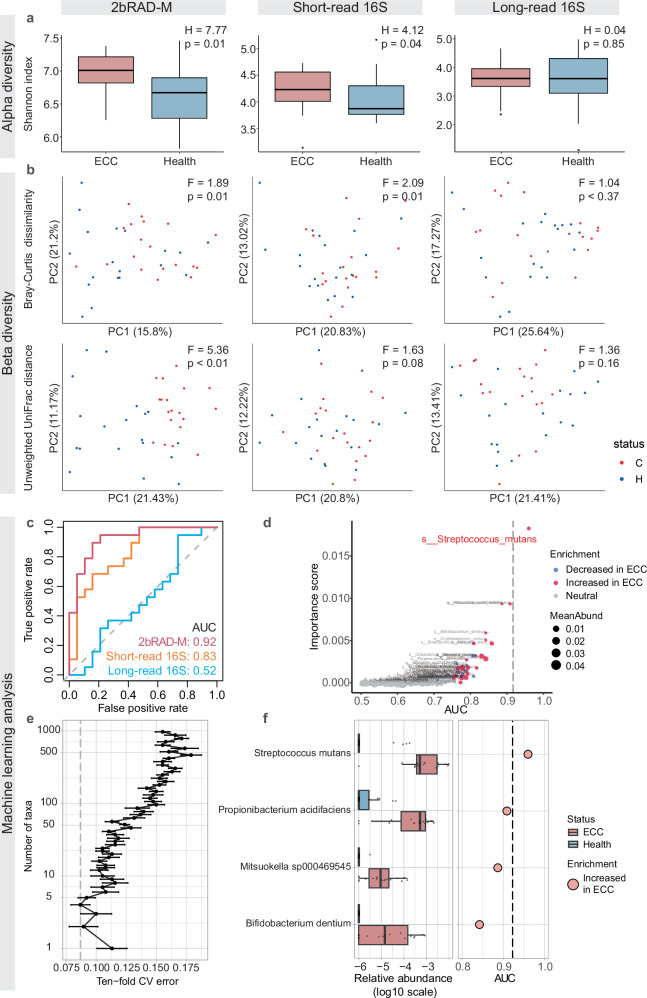
Sequencing of ECC saliva samples using 2bRAD-M, long-read 16S sequencing, and short-read 16S sequencing, with diversity analysis and disease classification results based on species-level profiles. Diversity analysis encompasses **a** alpha diversity indices and **b** beta diversity disparities discerned between healthy (blue) and ECC-affected (red) samples. The Kruskal–Wallis test was used to evaluate alpha diversity differences, and PERMANOVA was used for beta diversity. The H and F values represent the test statistics, indicating the magnitude of the differences. **c** Classification performance of the Random Forest model using species profiles of the three sequencing strategies, assessed by the area under the ROC curve (AUC). **d** The scatter plot displays the relationship between the importance scores of different microbial species and their respective AUC values. Red dots indicate an increase in ECC, blue dots signify a decrease, and gray dots represent a neutral status. The size of the dots represents the mean abundance of these species. **e** Relationship between the number of variables in the 2bRAD-M model and its predictive performance (the error bar of each dot denotes the standard deviation). **f** The four most discriminant species in the predictive model are shown using a box plot. The left side of the graph shows the log10-transformed relative abundance of each species in the healthy or ECC group. AUC assesses the utility of each taxon as a potential ECC marker on the right side of the graph.

We performed diversity analyses and developed machine learning models to compare the effectiveness of three sequencing methods in differentiating ECC from healthy oral microbiomes. Our analyses revealed that both 2bRAD-M and 16S short-read sequencing effectively distinguish between the ECC-affected and healthy groups. Conversely, the long-read sequencing method was less successful at making this distinction. Specifically, alpha diversity analysis indicates a significant difference in the Shannon index between the two groups for both 2bRAD-M and 16S short-read sequencing, as confirmed by the Kruskal–Wallis test (*p* < 0.05) (Fig. 7a). Notably, the Shannon index was higher with 2bRAD-M, suggesting it may detect a broader array of species compared to the 16S method. We then performed beta diversity analysis to examine the ecological differences between oral microbiotas of ECC and healthy groups. 2bRAD-M outperforms other methods, as evidenced by significant between-sample disparities using both unweighted UniFrac distances and Bray–Curtis dissimilarities (Fig. 7b). 16S short-read data performed moderately well, distinguishing the two groups based on Bray–Curtis dissimilarities but not in unweighted UniFrac distances. In contrast, 16S long-read sequencing data exhibits poor performance. These findings highlight the superior ability of 2bRAD-M to capture the complexity of oral microbiomes, emphasizing its potential for enhancing ECC diagnostics.

Random forest models built from microbial abundance profiles were evaluated for the diagnosis of ECC. We measured the models’ effectiveness by the area under the receiver operating characteristic curve (AUC), which quantifies their ability to distinguish between ECC and ECC-free cases. The 2bRAD-M sequencing approach yielded the most accurate results (AUC = 0.92), surpassing 16S short-read sequencing (AUC = 0.83) and significantly outperforming 16S long-read sequencing, which showed minimal discrimination (AUC = 0.52) (Fig. 7c). Both the 2bRAD-M and 16S short-read sequencing models identified *Streptococcus mutans* as a key indicator for ECC. Notably, the 2bRAD-M model identified additional biomarkers, underscoring its ability to reveal more nuanced disease state indicators (Fig. 7d). The 2bRAD-M model achieved peak performance using only the four most discriminative bacterial species as predictors, validated by rigorous 10-fold cross-validation that highlights their exceptional diagnostic power (Fig. 7e). Alongside *Streptococcus mutans*, this cohort includes *Propionibacterium acidifaciens*, *Mitsuokella sp000469545*, and *Bifidobacterium dentium*. Notably, the genus *Mitsuokella*, along with the other three species, has been independently associated with dental caries pathogenesis^37–40^ (Fig. 7F). Although 2bRAD-M further extended this analysis to fungi, identifying species such as *Candida albicans*, *Malassezia restricta*, and *Vanrija humicola* across multiple hosts, none exhibited significant differential abundance (Fig. S15). Collectively, the high-precision profiling results provided by 2bRAD-M are crucial for constructing accurate machine learning models and identifying species-resolved markers related to ECC.

## Discussion

Our study underscored the robust capabilities of 2bRAD-M sequencing in the precise delineation of microbial communities within HoC samples. We implemented 2bRAD-M sequencing and benchmarked its performance against conventional metagenomic methods using mock communities, saliva samples, and oral cancer specimens. The outcomes highlighted the effectiveness of 2bRAD-M in the precise microbial identification and abundance estimation within samples containing more than 90% host DNA. In the examination of salivary microbiome oscillations, 2bRAD-M demonstrated not only its ability to yield profiles congruent with those obtained via WMS with reduced sequencing effort but also its ability to discern minor temporal alterations in microbial communities. A cohort analysis effectively distinguished the discriminative microbiota of individuals afflicted with ECC from that of healthy counterparts, underscoring its utility in identifying critical microbial indicators in HoC samples. The study concludes that 2bRAD-M is a high-performance, cost-effective sequencing method for analyzing microbial communities in HoC samples, offering profound insights into the intricate dynamics of host-microbe interactions. These advantages position 2bRAD-M as a powerful tool for large-scale studies of chronic diseases and microbial dynamics, paving the way for the discovery of biomarkers for disease risk stratification and prevention through the profiling of thousands of samples.

Host depletion strategies remain a challenge for analyzing HoC samples, highlighting the importance of the rational development of 2bRAD-M in this study. Current host depletion strategies generally exploit structural or genomic differences between host and microbial cells; however, each approach has its limitations^5^. Pre-extraction methods employ selective cell lysis buffers to process host cells in the sample, followed by the breakdown of host DNA using DNase or PMA. Nonetheless, systematic evaluations indicate that PMA’s efficacy is considerably constrained by factors such as initial biomass, sample type, and chemical environment, which can potentially distort the true profile of microbial communities in complex setting samples^41^. Moreover, almost all pre-extraction methods, including those modified schemes based on PMA and other newly proposed methods like MEM, require immediate processing of fresh samples to prevent multiple freeze-thaw cycles that significantly damage microbial integrity in clinical samples^6^. These protocols also involve multiple lysis and centrifugation steps, which inevitably lead to DNA loss of both host and microbial cells, thus reducing their effectiveness in processing HoC samples^7,42^.

Post-extraction methods aim to separate microbial DNA from host contamination, for instance, by employing selective amplification with DNA probes that specifically bind to microbial or host DNA^43^, or by exploiting differences in DNA methylation between eukaryotes and prokaryotes through the use of DNA-binding proteins or methylation-sensitive restriction enzymes to enrich microbial DNA^44^. The application conditions for post-extraction methods are even more stringent: One limitation of using DNA probes is the prerequisite knowledge of the microbial DNA sequences to be enriched. Methods employing hybridization of probes to host DNA are not suitable for HoC samples because their effectiveness is greatly reduced by the vast and complex nature of host DNA; Moreover, only a few microbial methylomes are well-defined, and some prokaryotic methylation patterns are highly similar to those of eukaryotes, severely restricting the application of methylation-based separation methods^45^. In contrast, the 2bRAD-M method offers several advantages over these approaches, including being pretreatment-free and requiring minimal processing while simultaneously producing species-level bacterial, archaeal, and fungal profiles. Unlike host depletion strategies, 2bRAD-M also retains host DNA information in the profile, which can help studies on host-microbe interactions in clinical samples.

The benchmarking analysis of the mock samples revealed that 16S rRNA sequencing exhibited pronounced false-positive rates, erroneously identifying a substantial array of microbial taxa absent from the empirical ground truth. Notably, even the deployment of the advanced 5R 16S technique fell short in providing dependable species-level profiles against a backdrop of high host DNA content. This phenomenon may be attributed to “off-target amplification” when sequencing HoC samples with the 16S method, resulting from marker-gene primers binding to the host DNA (e.g., mitochondrial DNA in humans). It was reported that amplified human or mouse DNA sequences were erroneously clustered and assigned to bacterial taxa^3^. In our study, the relative abundance of *E. flexneri* significantly increases with the augmented proportion of host DNA, potentially indicating a proclivity for amplification of *Escherichia*.

Regarding the ECC saliva samples in our study, the long-read 16S rRNA sequencing failed to differentiate between disease and health. A recent study highlighted that full-length 16S rRNA gene sequencing with PacBio, compared to Illumina short-read sequencing, enhanced the taxonomic resolution in human microbiome samples^46^. Despite nominally detecting more species than short-read 16S (191 vs. 146), long-read 16S sequencing failed to differentiate ECC disease states. This diagnostic limitation arises from a fundamental constraint of third-generation sequencing: inherently higher error rates under low-biomass conditions. These random errors cause false contigs during assembly and significantly reduce the recovery of high-quality reads after denoising procedures^47^. Consequently, only 38.4% of raw reads passed through the denoising pipeline. In comparison, 2bRAD-M’s non-amplicon design inherently bypasses these limitations, delivering robust and artifact-free microbial profiling in host-rich diagnostic applications.

We acknowledge the limitations of this study. The validation of 2bRAD-M’s performance in HoC samples was primarily based on saliva and tumor specimens. Large-scale validation across broader anatomical sites with high host DNA content—such as cervicovaginal swabs, intestinal biopsies, blood, and other tissue types—remains essential to solidify the generalizability of these preliminary findings in diverse host-dominated ecosystems. For widespread implementation, key challenges remain, including the need for continued expansion of reference databases to enhance taxonomic resolution and rigorous validation in each new sample matrix to establish clinical utility. Additionally, as a reference-dependent method, 2bRAD-M’s performance is highly contingent on database selection. In this study, GTDB was uniformly applied to both 2bRAD-M and 16S data types. By contrast, WMS profiling tools (e.g., MetaPhlAn4) typically utilize distinct reference databases that are not customized for GTDB alignment, leading to potential discrepancies during cross-method comparisons. To minimize such confounders, we processed all sequencing data through the 2bRAD-M pipeline with GTDB, ensuring consistent bioinformatic benchmarking.

## Methods

### Mock samples preparation

The mock sample employed for evaluating the efficacy of the 2bRAD-M sequencing approach, in comparison with other sequencing techniques, was a 20-species uniform genomic mix, designated MSA 1002 (ATCC). The selected mock comprises genomic DNA prepared from fully sequenced, characterized, and authenticated “ATCC Genuine Cultures”. These cultures were judiciously chosen to represent a spectrum of pertinent phenotypic and genotypic characteristics, including Gram staining reaction, guanine-cytosine (G+C) content, genome size, and spore-forming capability. Subsequently, experimental conditions were meticulously devised to mimic the high complexity human microbiome samples, which were formulated to contain either 90% or 99% human DNA, with two replicates generated for each mixture.

### Diurnal saliva sample characteristics

For this experiment, 16 participants were randomly recruited from the Qingdao branch of Shanghai OE Biotech Co., Ltd. This study received ethical approval from the Ethics Committee of Qingdao Municipal Hospital of Stomatology (certificate number: 2022KQYX030). The same professional dentist performed all sample collections to minimize exogenous contamination in the samples. To this end, participants were instructed not to eat or drink for two hours before the sampling time. The exclusion criteria for participants included the use of antibiotics or medication in the previous month. Samples from eight participants, for whom the clean data accounted for more than 70% of the raw data according to the 2bRAD-M analysis, were selected for further WMS and 16S rRNA sequencing.

### Description of oral cancer specimens

Oral cancer specimens were obtained from a southern Chinese cohort at Queen Mary Hospital under IRB approval UW 15-239 (The University of Hong Kong/Hospital Authority Hong Kong West Cluster). Before enrollment, all participants provided written informed consent using IRB-approved documentation (dated 21 March 2015). Specimen collection and handling protocols were strictly adhered to the Declaration of Helsinki and ICH-GCP guidelines. Clinicopathological and follow-up data were retrospectively collected from hospital records. Fresh tumor tissues were snap-frozen in liquid nitrogen and stored at −80 °C until extraction.

### ECC saliva sample characteristics

Between March and June 2019, an oral epidemiological survey was conducted among 5-year-old children across 13 randomly selected kindergartens in Qingdao, China. This study received ethical approval from the Ethics Committee of Qingdao Municipal Hospital of Stomatology (certificate number: 2022KQYX031), and informed consent was obtained from all participants or their guardians, detailing oral examination procedures, sample collection, environmental factor gathering, and the use of data in subsequent research and publications. Informed consent was obtained from the participants. All participants gave written consent for their clinical details to be published. A total of 63 children participated in the study, comprising 34 males and 29 females. Based on the decayed, missing, and filled surfaces (dmfs) index, participants were classified into two groups: a high-caries group (*n* = 32) with severe early childhood caries (dmfs ≥6) and a healthy group (*n* = 31) with no caries (dmfs = 0). Eligibility criteria included no antibiotic use within three months before the examination, a dentition consisting entirely of primary teeth with at least 20 teeth present, normal oral mucosa in color and texture, no congenital malformations or systemic diseases, and no orthodontic appliances or accessories worn.

### Saliva sample collection

Participants were instructed to tilt their heads downward, slightly open their mouths, and rest their lower lip against the opening of a 15 mL sterile centrifuge tube, facilitating the collection of unstimulated saliva. The collection period lasted approximately 5 min, during which participants were asked to expectorate saliva into the collection tube. Approximately 5 ml of saliva was collected from each participant per session.

### DNA extraction, whole metagenomic shotgun sequencing, 16S rRNA short-read and long-read sequencing

Sample collection tubes were thawed on ice and then centrifuged thoroughly. Adhering to the manufacturer’s guidelines with minor adjustments, bacterial genomic DNA was extracted from saliva samples using the Tissue and Blood DNA Isolation kit (Qiagen). Tumor DNA was extracted from fresh frozen tumor samples using Cetyltrimethylammonium Bromide (CTAB). The tissue block was ground with liquid nitrogen, and 50 mg was transferred to a 2.0 mL centrifuge tube containing 1 mL of CTAB lysis buffer. The mixture was incubated at 65 °C with occasional mixing until fully lysed. The lysate was then centrifuged, and the supernatant was extracted with phenol (pH 8.0): chloroform: isoamyl alcohol (25:24:1), followed by chloroform: isoamyl alcohol (24:1). DNA was precipitated with isopropanol at −20 °C, centrifuged, washed twice with 75% ethanol, and air-dried. The DNA was dissolved in ddH2O, incubated at 55–60 °C if necessary, and treated with RNase A at 37 °C for 15 min.

For the mock samples, we attempted to sequence the 16S rRNA gene’s highly variable regions V2-V3, V4-V5, and five regions from V2 to V8 (termed 5R 16S) and compared the results. The 5R 16S rRNA sequencing approach amplifies regions covering approximately 68% of the full length of the 16S sequence, significantly enhancing the coverage and resolution of bacterial species detection, making it particularly suitable for low-biomass microbial sample analysis.

qPCR was performed on a Roche LightCycler 480 instrument. Reactions (10 μL total volume) contained: 5 μL 2× TB Green II (Takara), 0.2 μL each forward/reverse primer (10 μM), 0.2 μL 50× ROX, and 1 μL template DNA. Bacterial abundance was quantified using the V4-V5 region of the 16S rRNA gene with universal primers 515F/907R. *Porphyromonas endodontalis* abundance was quantified using species-specific primers (Forward: 5’-CTATATTCTTCTTTCTCCGCATGGAGGAGG-3’; Reverse: 5’-GCATACCTTCGGTCTCCTCTAGCATAT-3’). Thermal cycling: 95 °C for 30 s (initial denaturation); 40 cycles of 95 °C for 5 s, 55 °C for 30 s, 72 °C for 30 s; followed by melting curve analysis. Standard curves were generated from 10-fold serial dilutions of plasmid DNA.

For the diurnal saliva samples, considering cost issues and the current state of 16S rRNA technology application, we employed the most commonly used sequencing approach by amplifying the V3V4 highly variable regions of the 16S rRNA gene using the primer pair (338F/806R). PCR amplification for each sample was performed in triplicate, using 15 μl of Phusion® High-Fidelity PCR Master Mix (New England Biolabs), 0.2 μM of both forward and reverse primers, and approximately 10 ng of template DNA.

For long-read 16S rRNA sequencing, total DNA was extracted from the ECC saliva samples. Using the full-length 16S primers (i.e., 27F and 1492R), barcode-tagged specific primers were synthesized for PCR amplification. The PCR products were then purified, quantified, and normalized to prepare the sequencing library (SMRT Bell). Once the library was constructed, it underwent quality control checks. Libraries that passed QC were sequenced using the PacBio Sequel system. The PacBio Sequel generates data in BAM format, which is then exported as CCS files using the SMRT Link analysis software. The data for different samples, identified by barcode sequences, are converted into FASTQ format for further analysis. All sequences were processed according to the standard QIIME2 (2023.2) pipeline for preprocessing and downstream bioinformatics analysis. The clustering of ASVs was performed using the DADA2 plugin provided by QIIME2.

Both mock community samples (*N* = 6) and diurnal saliva samples (*N* = 32) were analyzed with whole metagenomics sequencing (WMS) using the MGI DNBSEQ-T7RS platform. Clean reads were produced and taxonomically characterized using MetaPhlAn4 and Bracken2 with standard settings.

### 2bRAD-M sequencing

The 2bRAD-M library preparation, with minor modifications from the original method^48^, was initiated by digesting genomic DNA (1 pg to 200 ng) with 4 U BcgI (NEB) in a 15-μl reaction at 37 °C for three hours. 5 μl of the product was assessed on a 1% agarose gel for complete digestion. The ligation reaction, conducted at 16 °C overnight, consisted of a 20-μl reaction mixture comprising enzyme-digested DNA, 2 μl of 1× T4 ligase buffer, 1 μl of adapters, and 800 U of T4 DNA ligase (NEB).

Ligation products underwent PCR amplification in a 40-μl reaction including 7 μl of DNA, 0.1 μM of each Illumina-specific primer (Primer1 and Primer2), 0.3 mM dNTPs, 1× Phusion HF buffer, and 0.4 U Phusion DNA polymerase (NEB). The PCR, using a DNA Engine Tetrad 2 thermal cycler (Bio-Rad), involved 16–28 cycles at 98 °C for 5 s, 60 °C for 20 s, and 72 °C for 10 s, with a final extension at 72 °C for 10 min. The desired band (~100 bp) was excised from an 8% polyacrylamide gel, and DNA was eluted into nuclease-free water for 12 h at 4 °C. Barcode introduction was performed in a secondary 40-μl PCR containing 50 ng of extracted DNA, 0.2 μM of each barcode-bearing primer (Primer1 and Primer3), 0.6 mM dNTPs, 1× Phusion HF buffer, and 0.8 U Phusion DNA polymerase for seven cycles. The amplified products were purified using the QIAquick PCR Purification Kit (Qiagen) and sequenced on the Illumina HiSeq platform.

### Update of the 2bRAD-M Tag database

To identify taxa-specific molecular tags, we constructed an updated 2bRAD-M tag database (2b-Tag-DB) through in silico restriction digestion of microbial genomes sourced from the Genome Taxonomy Database (GTDB; release r202) and EnsemblFungi (https://fungi.ensembl.org/index.html). This comprehensive genome dataset comprised 259,388 microbial genomes (254,090 bacteria, 982 fungi, and 4316 archaea), collectively representing 48,475 distinct species. Using the type IIB restriction enzyme BcgI, we generated iso-length (32 bp) 2bRAD tags from these genomic sequences. The final database contained 361,631,938 unique BcgI-digested tags (averaging 1,395.4 tags per species genome), providing a high-resolution reference for taxonomic assignment.

### Computational workflow of 2bRAD-M

The 2bRAD-M computational pipeline aims to reconstruct high-resolution taxonomic profiles from iso-length restriction fragments while tackling challenges caused by high levels of host DNA contamination^12^. First, sequencing reads are mapped to a precomputed 2b-Tag-DB—a reference database of species-specific 2bRAD markers created from 259,388 microbial genomes (including bacteria, archaea, and fungi) using Type IIB restriction enzymes (e.g., BcgI). Raw sequencing reads undergo quality control and are aligned to this database, with initial taxonomic assignments based on species-specific marker matches. Residual host DNA interference is reduced by excluding reads that match precomputed host-derived motifs. To mitigate the impact of potential contamination, a bioinformatic decontamination step is applied utilizing sequencing data from negative controls. This involves calculating and subtracting a normalized proportion of contaminating reads ($D=T\times \frac{T}{N}$, where T and N are the total reads in the sample and negative control, respectively) that are likely attributable to the laboratory background. Additionally, a *G*-score threshold ($G=\sqrt{S\times t}$, where *S*= coverage and *t* = marker specificity) is applied, discarding taxa below a certain threshold (Default $G < 5$). To improve resolution, a dynamic, sample-specific database is created by adding more markers from candidate taxa identified in the sample, which increases resolution and decreases false positives. Species abundance is then calculated as the mean coverage of taxon-specific markers normalized by their expected genome-wide count, adjusting for differences in genome size and marker density. Detailed methodological information, including computational scripts, parameter settings, and implementation instructions, is available in the 2bRAD-M GitHub repository (https://github.com/shihuang047/2bRAD-M).

### Metagenomic profiling workflow

For comprehensive taxonomic characterization of shotgun metagenomic datasets, we employed analytical approaches that integrated three methods: MetaPhlAn4, Kraken2^49^, and Bracken. In the future, such analysis will be developed into a version that can be incorporated in our EasyMetagenome pipeline^50,51^.

MetaPhlAn4 (v4.0.6) uses a clade-specific marker-gene alignment strategy, employing a precompiled database of genetic markers curated for microbial clade discrimination. Reads were aligned to these marker sequences using Bowtie2 (v2.5.0) for accurate taxonomic assignment and abundance estimation. Analyses were performed with the mpa_vOct22_CHOCOPhlAnSGB_202212 database, which includes species-level genomic bins (SGBs) for better resolution of uncultivated microbes. The following command-line implementation was used:

metaphlan ./clean.fq.gz --bowtie2out genome.bz2 --nproc 16 --input_type fastq -o genome.txt --bowtie2db /lustre1/g/aos_shihuang/databases/metaphlan4_db

Kraken2 (v2.1.2) functions as a k-mer-based taxonomic classifier, employing exact 35-bp k-mer matches to assign sequences to the lowest common ancestor (LCA) within a hierarchically organized reference database. This database was assembled from all complete bacterial genomes stored in the Kraken database (minikraken2_v1_8GB), ensuring broad coverage of microbial diversity. To improve specificity, a minimum abundance threshold of 0.01 (default) was applied to filter out low-confidence taxa. The following command-line implementation was executed:

kraken2 --db /lustre1/g/aos_shihuang/tools/kraken2-standard-db/kraken_database/ --threads 12 --report genome.report --output genome.output --paired ../genome_1.clean.fq.gz ../genome_2.clean.fq.gz

Bracken (v2.5.0) employs the read classification results from standard Kraken to perform a Bayesian re-estimation of taxonomic abundances. This process effectively addresses common false-positive issues associated with Kraken and naturally accounts for variations in genome length. The kraken-filter tool was applied to filter raw classifications at a threshold of 0.01. The specific Bracken command used is provided below:

bracken -d /lustre1/g/aos_shihuang/tools/kraken2-standard-db/kraken_database/ -i genome.report -o genome.bracken -w genome.bracken.report -r 150 -l S -t 10

### Evaluation metrics: precision, recall, AUPR, L2 similarity, and Pearson coefficient

To gauge overall performance, we employed precision and recall for the accuracy of microbial identification and L2 distance to assess the effectiveness of abundance estimations in samples. Precision is determined by the ratio of correctly identified species to the overall species identified by the technique, and recall measures the proportion of correctly identified species relative to all species present in the sample. Adjusting the threshold for classifying a species as a true positive varies the precision and recall outcomes. Plotting these metrics, with recall on the x-axis and precision on the y-axis, creates the Precision–Recall (PR) curve. The area under this curve, known as the Area Under the Precision–Recall Curve (AUPR), quantifies the average performance of the model across various decision thresholds in terms of precision and recall. To assess the effectiveness of the abundance estimation, the L2 distance was calculated between the abundance profile of the ground truth and those produced by various metagenomic sequencing methods at specific taxonomic levels (species and genus). For a clearer performance comparison, L2 similarity was determined by one minus the L2 distance. Furthermore, in the Pearson correlation analysis, when a species was uniquely identified by one method, its abundance was set to zero in the computation of the Pearson correlation coefficient.

### Diagnosis model of ECC samples

A random forest model was trained to determine disease status based on taxonomic profiles and was then evaluated using the area under the Receiver Operating Characteristic (ROC) curve. The model was implemented using default parameters from the R package “randomForest” (ntree = 5000, errortype = “oob”, n.oob = 10, nfolds = 10). The performance of the microbiome-based model was further assessed through 10-fold cross-validation using species profiles. According to the “rfcv” function in the random forest package, the top four ranked important taxa facilitated a considerably accurate classification of ECC status. A comprehensive method description is provided in the supplementary information.

## Supplementary information

Supplementary information

## Data Availability

The sequencing data of 2bRAD-M, WMS, next-generation 16S, and third-generation sequencing for Mock samples (ATCC MSA 1002 combined with 90% and 99% human DNA) and real HoC samples (diurnal saliva samples, oral cancer specimens, and ECC saliva samples) were stored at the Sequence Read Archive (SRA): https://www.ncbi.nlm.nih.gov/bioproject/PRJNA1131785.

## Code availability

The 2bRAD-M computational pipeline and its associated database files are publicly accessible on GitHub at https://github.com/shihuang047/2bRAD-M. The 2bRAD-M computational pipeline is distributed under the MIT license. All source data and codes for the generation of figures and tables in the manuscript can be downloaded from: https://github.com/jiangys30/HoC_project.git.

## References

[1] Marotz, C. A. et al. Improving saliva shotgun metagenomics by chemical host DNA depletion. *Microbiome* **6**, 42 (2018). 29482639 10.1186/s40168-018-0426-3 PMC5827986

[2] Sepich-Poore, G. D. et al. Cancer’s second genome: microbial cancer diagnostics and redefining clonal evolution as a multispecies process: humans and their tumors are not aseptic, and the multispecies nature of cancer modulates clinical care and clonal evolution: humans and their tumors are not aseptic, and the multispecies nature of cancer modulates clinical care and clonal evolution. *Bioessays* **44**, e2100252 (2022). 35253252 10.1002/bies.202100252 PMC10506734

[3] Bedarf, J. R. et al. Much ado about nothing? Off-target amplification can lead to false-positive bacterial brain microbiome detection in healthy and Parkinson’s disease individuals. *Microbiome* **9**, 75 (2021. 33771222 10.1186/s40168-021-01012-1 PMC8004470

[4] Knight, R. et al. Best practices for analysing microbiomes. *Nat. Rev. Microbiol* **16**, 410–422 (2018). 29795328 10.1038/s41579-018-0029-9

[5] Shi, Y., Wang, G., Lau, H. C. & Yu, J. Metagenomic sequencing for microbial DNA in human samples: emerging technological advances. *Int. J. Mol. Sci.* **23**, 2181 (2022). 35216302 10.3390/ijms23042181 PMC8877284

[6] Liu, F. et al. Systematic evaluation of the viable microbiome in the human oral and gut samples with spike-in Gram+/- bacteria. *mSystems* **8**, e0073822 (2023). 36971593 10.1128/msystems.00738-22 PMC10134872

[7] Hasan, M. R. et al. Depletion of human DNA in spiked clinical specimens for improvement of sensitivity of pathogen detection by next-generation sequencing. *J. Clin. Microbiol.* **54**, 919–927 (2016). 26763966 10.1128/JCM.03050-15 PMC4809942

[8] Xu, C., Bian, C., Lam, R., Dong, A. & Min, J. The structural basis for selective binding of non-methylated CpG islands by the CFP1 CXXC domain. *Nat. Commun.* **2**, 227 (2011). 21407193 10.1038/ncomms1237 PMC3072069

[9] Oyola, S. O. et al. Efficient depletion of host DNA contamination in malaria clinical sequencing. *J. Clin. Microbiol.* **51**, 745–751 (2013). 23224084 10.1128/JCM.02507-12 PMC3592063

[10] Liu, G. H. et al. Epigenetic segregation of microbial genomes from complex samples using restriction endonucleases HpaII and McrB. *PLoS ONE* **11**, e0146064 (2016). 26727463 10.1371/journal.pone.0146064 PMC4699840

[11] Groussin, M., Mazel, F. & Alm, E. J. Co-evolution and Co-speciation of Host-Gut Bacteria Systems. *Cell Host Microbe* **28**, 12–22 (2020). 32645351 10.1016/j.chom.2020.06.013

[12] Sun, Z. et al. Species-resolved sequencing of low-biomass or degraded microbiomes using 2bRAD-M. *Genome Biol.* **23**, 36 (2022). 35078506 10.1186/s13059-021-02576-9 PMC8789378

[13] Andrews, K. R., Good, J. M., Miller, M. R., Luikart, G. & Hohenlohe, P. A. Harnessing the power of RADseq for ecological and evolutionary genomics. *Nat. Rev. Genet* **17**, 81–92 (2016). 26729255 10.1038/nrg.2015.28 PMC4823021

[14] Sanz, M. et al. Role of microbial biofilms in the maintenance of oral health and in the development of dental caries and periodontal diseases. Consensus report of group 1 of the Joint EFP/ORCA workshop on the boundaries between caries and periodontal disease. *J. Clin. Periodontol.* **44**, S5–S11 (2017). 28266109 10.1111/jcpe.12682

[15] Sabharwal, A. et al. The salivary microbiome of diabetic and non-diabetic adults with periodontal disease. *J. Periodontol.* **90**, 26–34 (2019). 29999529 10.1002/JPER.18-0167

[16] Kageyama, S. et al. Characteristics of the salivary microbiota in patients with various digestive tract cancers. *Front. Microbiol.* **10**, 1780 (2019). 31428073 10.3389/fmicb.2019.01780 PMC6688131

[17] Lewy, T. et al. Oral microbiome in HIV-infected women: shifts in the abundance of pathogenic and beneficial bacteria are associated with aging, HIV load, CD4 count, and antiretroviral therapy. *AIDS Res Hum. Retroviruses* **35**, 276–286 (2019). 29808701 10.1089/aid.2017.0200 PMC6909750

[18] Wallen, Z. D. et al. Metagenomics of Parkinson’s disease implicates the gut microbiome in multiple disease mechanisms. *Nat. Commun.* **13**, 6958 (2022). 36376318 10.1038/s41467-022-34667-x PMC9663292

[19] Bhattarai, K. R., Kim, H. R. & Chae, H. J. Compliance with saliva collection protocol in healthy volunteers: strategies for managing risk and errors. *Int. J. Med. Sci.* **15**, 823–831 (2018). 30008593 10.7150/ijms.25146 PMC6036086

[20] Mivehchi, H. et al. Exploring the role of oral bacteria in oral cancer: a narrative review. *Discov. Oncol.* **16**, 242 (2025). 40009328 10.1007/s12672-025-01998-2 PMC11865422

[21] Parks, D. H. et al. GTDB: an ongoing census of bacterial and archaeal diversity through a phylogenetically consistent, rank normalized and complete genome-based taxonomy. *Nucleic Acids Res.* **50**, D785–D794 (2022). 34520557 10.1093/nar/gkab776 PMC8728215

[22] Yates, A. D. et al. Ensembl Genomes 2022: an expanding genome resource for non-vertebrates. *Nucleic Acids Res.* **50**, D996–D1003 (2022). 34791415 10.1093/nar/gkab1007 PMC8728113

[23] Nejman, D. et al. The human tumor microbiome is composed of tumor type-specific intracellular bacteria. *Science* **368**, 973 (2020). 32467386 10.1126/science.aay9189 PMC7757858

[24] Bolyen, E. et al. Reproducible, interactive, scalable and extensible microbiome data science using QIIME 2. *Nat. Biotechnol.* **37**, 852–857 (2019). 31341288 10.1038/s41587-019-0209-9 PMC7015180

[25] Blanco-Míguez, A. et al. Extending and improving metagenomic taxonomic profiling with uncharacterized species using MetaPhlAn 4. *Nat. Biotechnol.* **41**, 1633–1644 (2023). 36823356 10.1038/s41587-023-01688-w PMC10635831

[26] Lu, J., Breitwieser, F. P., Thielen, P. & Salzberg, S. L. Bracken: estimating species abundance in metagenomics data. *PeerJ Comput. Sci.* **3**, e104 (2017). 40271438 10.7717/peerj-cs.104 PMC12016282

[27] Ye, S. H., Siddle, K. J., Park, D. J. & Sabeti, P. C. Benchmarking metagenomics tools for taxonomic classification. *Cell* **178**, 779–794 (2019). 31398336 10.1016/j.cell.2019.07.010 PMC6716367

[28] Saito, T. & Rehmsmeier, M. The precision-recall plot is more informative than the ROC plot when evaluating binary classifiers on imbalanced datasets. *PLoS ONE* **10**, e0118432 (2015). 25738806 10.1371/journal.pone.0118432 PMC4349800

[29] Amos, G. C. A. et al. Developing standards for the microbiome field. *Microbiome* **8**, 98 (2020). 32591016 10.1186/s40168-020-00856-3 PMC7320585

[30] Quast, C. et al. The SILVA ribosomal RNA gene database project: improved data processing and web-based tools. *Nucleic Acids Res.* **41**, D590–D596 (2013). 23193283 10.1093/nar/gks1219 PMC3531112

[31] Zhu, Q. Y. et al. Phylogeny-aware analysis of metagenome community ecology based on matched reference genomes while bypassing taxonomy. *mSystems* **7**, e0016722 (2022). 35369727 10.1128/msystems.00167-22 PMC9040630

[32] Hu, Y. et al. Diurnal and eating-associated microbial patterns revealed via high-frequency saliva sampling. *Genome Res.* **32**, 1112–1123 (2022). 35688483 10.1101/gr.276482.121 PMC9248889

[33] Takayasu, L. et al. Circadian oscillations of microbial and functional composition in the human salivary microbiome. *DNA Res.* **24**, 261–270 (2017). 28338745 10.1093/dnares/dsx001 PMC5499806

[34] Teng, F. et al. Prediction of early childhood caries via spatial-temporal variations of oral microbiota. *Cell Host Microbe* **18**, 296–306 (2015). 26355216 10.1016/j.chom.2015.08.005

[35] Huang, S. et al. Predictive modeling of gingivitis severity and susceptibility via oral microbiota. *ISME J.* **8**, 1768–1780 (2014). 24646694 10.1038/ismej.2014.32 PMC4139724

[36] Casamassimo, P. S., Thikkurissy, S., Edelstein, B. L. & Maiorini, E. Beyond the dmft: the human and economic cost of early childhood caries. *J. Am. Dent. Assoc.* **140**, 650–657 (2009). 19491160 10.14219/jada.archive.2009.0250

[37] Obata, J., Fujishima, K., Nagata, E. & Oho, T. Pathogenic mechanisms of cariogenic Propionibacterium acidifaciens. *Arch. Oral. Biol.* **105**, 46–51 (2019). 31254840 10.1016/j.archoralbio.2019.06.005

[38] Skelly, E. et al. Response of salivary microbiota to caries preventive treatment in Aboriginal and Torres Strait Islander Children. *J. Oral. Microbiol.* **12**, 1830623 (2020). 33149844 10.1080/20002297.2020.1830623 PMC7586720

[39] Becker, M. R. et al. Molecular analysis of bacterial species associated with childhood caries. *J. Clin. Microbiol.* **40**, 1001–1009 (2002). 11880430 10.1128/JCM.40.3.1001-1009.2002 PMC120252

[40] Okada, M. et al. PCR detection of Streptococcus mutans and S. sobrinus in dental plaque samples from Japanese pre-school children. *J. Med Microbiol.* **51**, 443–447 (2002). 11990497 10.1099/0022-1317-51-5-443

[41] Wang, Y. et al. Whole microbial community viability is not quantitatively reflected by propidium monoazide sequencing approach. *Microbiome* **9**, 17 (2021). 33478576 10.1186/s40168-020-00961-3 PMC7819323

[42] Ferretti, P. et al. Experimental metagenomics and ribosomal profiling of the human skin microbiome. *Exp. Dermatol.* **26**, 211–219 (2017). 27623553 10.1111/exd.13210

[43] Smith, M. et al. An in-solution hybridisation method for the isolation of pathogen DNA from human DNA-rich clinical samples for analysis by NGS. *Open Genomics J.* **5**, 18–29 (2012). 10.2174/1875693X01205010018 PMC3837216 24273626

[44] Barnes, H. E. et al. Selective microbial genomic DNA isolation using restriction endonucleases. *PLoS ONE* **9**, e109061 (2014). 25279840 10.1371/journal.pone.0109061 PMC4184833

[45] Bewick, A. J. et al. Diversity of cytosine methylation across the fungal tree of life. *Nat. Ecol. Evol.* **3**, 479–490 (2019). 30778188 10.1038/s41559-019-0810-9 PMC6533610

[46] Buetas, E. et al. Full-length 16S rRNA gene sequencing by PacBio improves taxonomic resolution in human microbiome samples. *BMC Genomics* **25**, 310 (2024). 38528457 10.1186/s12864-024-10213-5 PMC10964587

[47] Pearman, W. S., Freed, N. E. & Silander, O. K. Testing the advantages and disadvantages of short- and long- read eukaryotic metagenomics using simulated reads. *BMC Bioinform.* **21**, 220 (2020). 10.1186/s12859-020-3528-4 PMC7257156 32471343

[48] Wang, S., Meyer, E., McKay, J. K. & Matz, M. V. 2b-RAD: a simple and flexible method for genome-wide genotyping. *Nat. Methods* **9**, 808–810 (2012). 22609625 10.1038/nmeth.2023

[49] Wood, D. E., Lu, J. & Langmead, B. Improved metagenomic analysis with Kraken 2. *Genome Biol.* **20**, 257 (2019). 31779668 10.1186/s13059-019-1891-0 PMC6883579

[50] Yousuf, S. et al. Unveiling microbial communities with EasyAmplicon: A user‐centric guide to perform amplicon sequencing data analysis. *iMetaOmics* **1**, e42 (2024). 10.1002/imo2.42 PMC12806499 41676130

[51] Bai, D. et al. EasyMetagenome: A user‐friendly and flexible pipeline for shotgun metagenomic analysis in microbiome research. *iMeta* **4**, e70001 (2025). 10.1002/imt2.70001 PMC11865343 40027489

