# Supplementary material for: High-resolution microbiome analysis of host-rich samples using 2bRAD-M without host depletion
Source: NPJ Biofilms Microbiomes. 2025 Nov 28;11:223. doi: 10.1038/s41522-025-00851-2 (PMC12663593; doi:10.1038/s41522-025-00851-2)
Supplement: Supplementary file 1 — Supplementary information [file 41522_2025_851_MOESM1_ESM.pdf]

Supplementary Figure legends

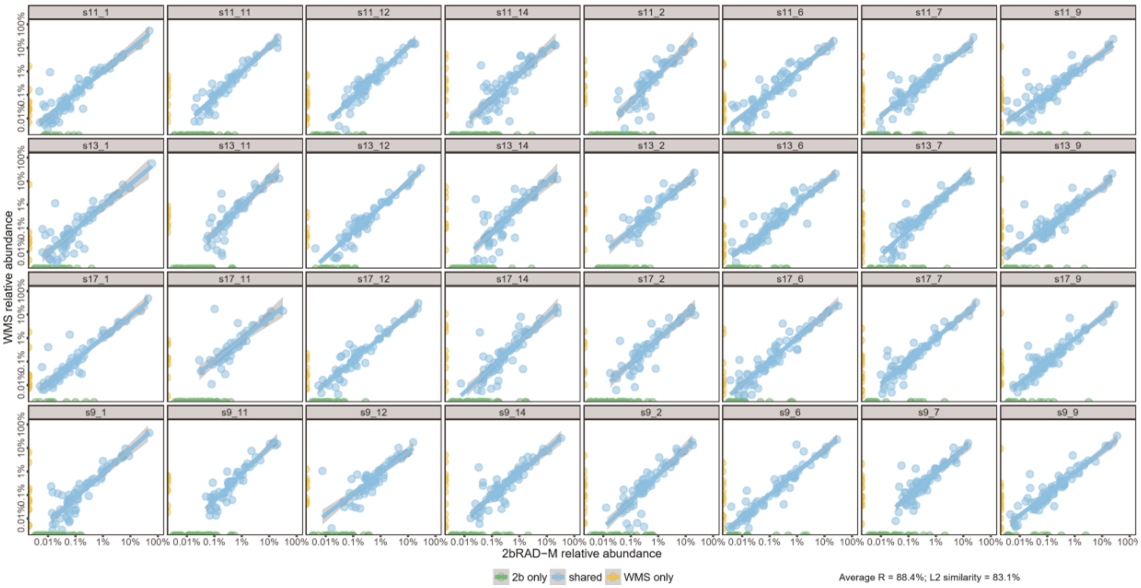

Fig. S1. Comparative analysis of genus-level taxonomic profiling results obtained from 2bRAD-M and WMS sequencing. "Average R" and "Average L2" labeled in the figure represent the respective average indices for all 32 samples. The gray box on top of each scatter plot displays the sample ID. Points on the coordinate axis represent unique features identified by the corresponding method, while shared features are indicated by light blue points in the white area of the plot. As shown in the figure, the profiles of 2bRAD-M and WMS are highly concordant.

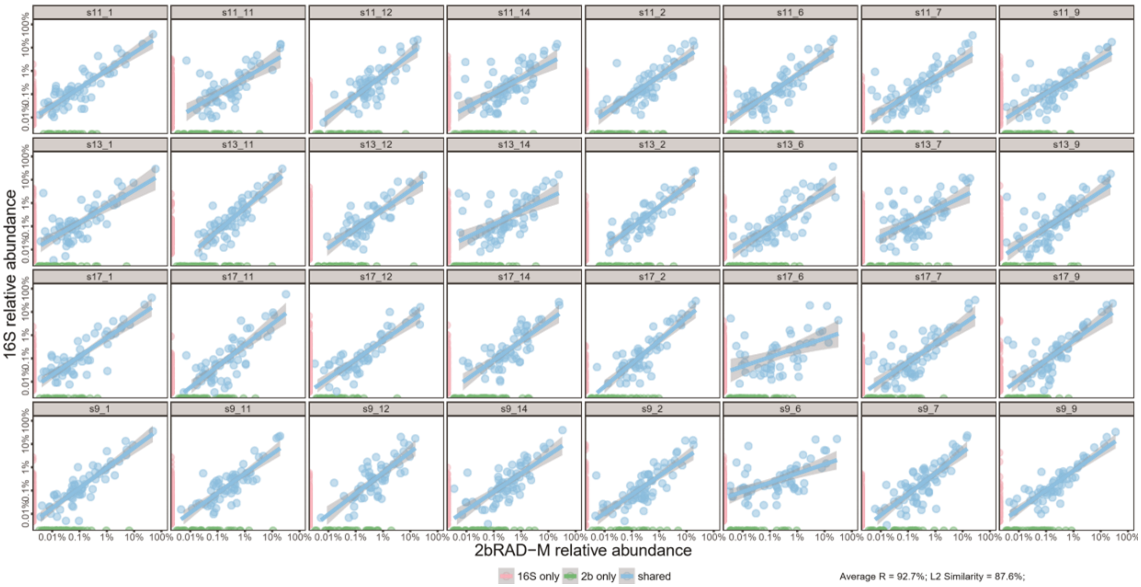

Fig. S2. Comparative analysis of genus-level taxonomic profiling results obtained

from 2bRAD-M and 16S rRNA sequencing. The green points on the x-axis represent genera that are only present in the profile obtained from 2bRAD-M, while the pink points on the y-axis represent genera that are unique to the 16S rRNA sequencing results. The taxonomic profiles obtained from 2bRAD-M and 16S rRNA sequencing exhibited a slightly lower concordance.

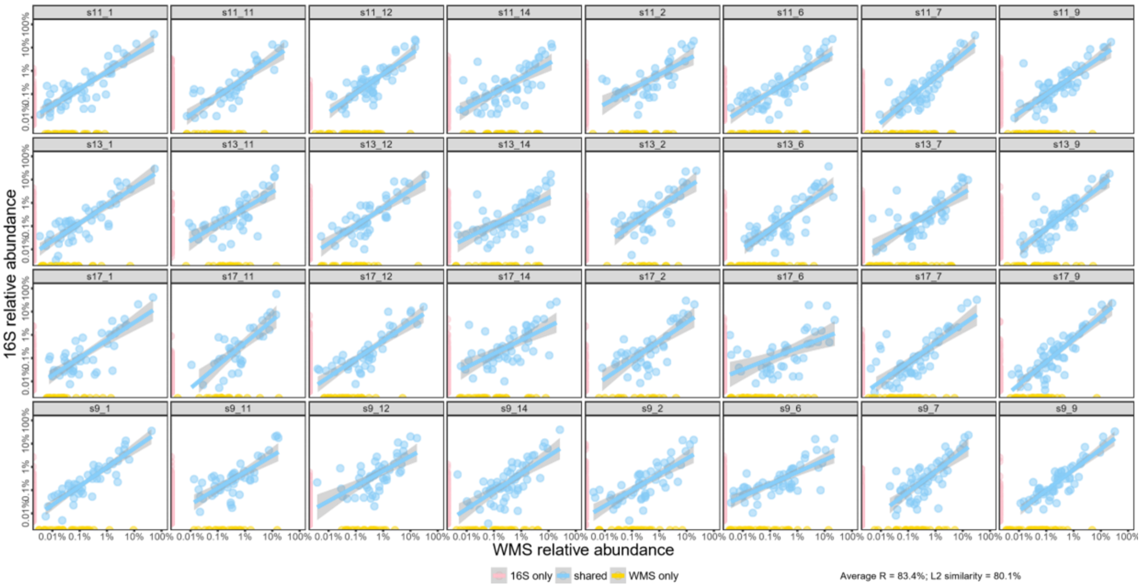

Fig. S3. Comparative analysis of genus-level taxonomic profiling results obtained from WMS and 16S rRNA sequencing. The yellow points on the x-axis represent genera that are only present in the WMS taxonomic profiling results, while the pink points on the y-axis represent genera that are unique to the 16S rRNA sequencing results. The light blue points in the middle represent genera that are detected by both sequencing methods. The image reflects that the similarity between 16S rRNA sequencing and WMS is slightly lower.

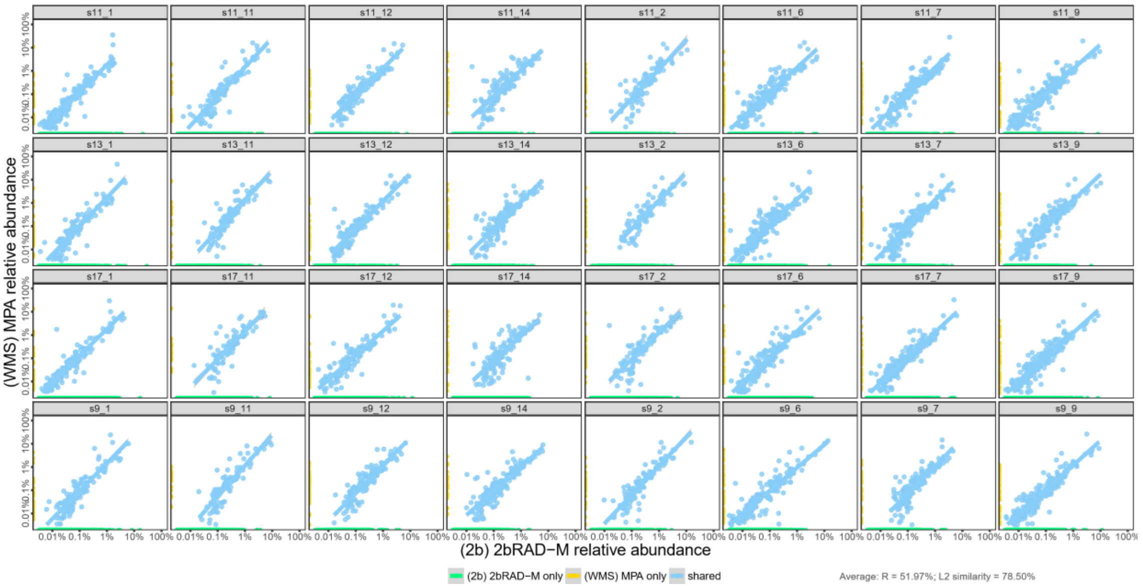

Fig. S4. Comparative analysis of species-level taxonomic profiling results obtained from 2bRAD-M and WMS based on overall divergence. The overall similarity of the corresponding taxonomic profiles at the species level obtained by the two methods is relatively low. (WMS) PMA: analyzing WMS data using MetaPhlAn4; (2b) 2bRAD-M: analyzing 2bRAD-M sequencing data with 2bRAD-M computational pipeline.

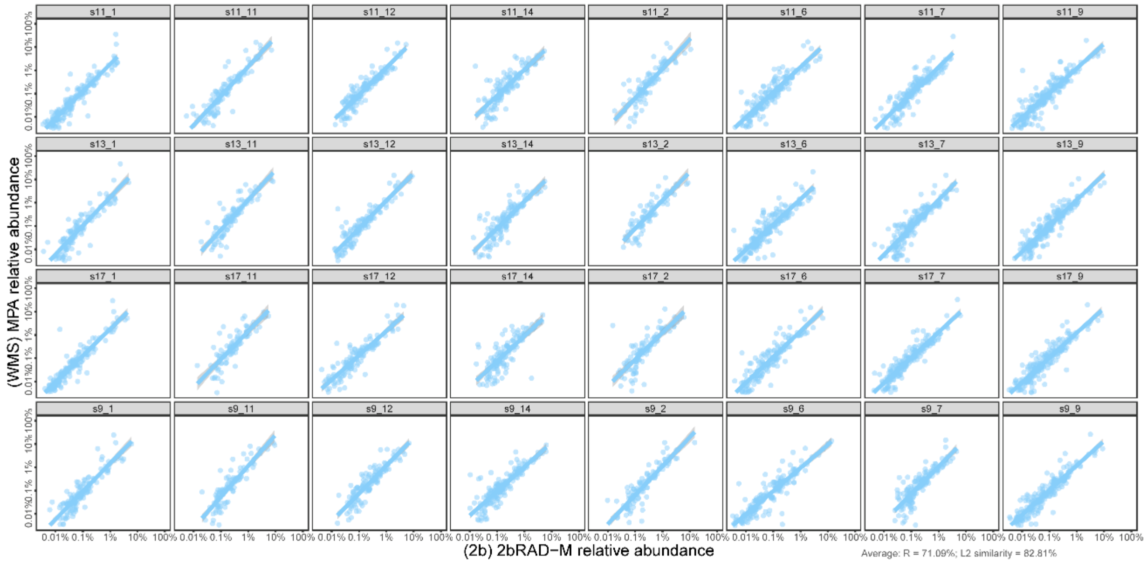

Fig. S5. Comparative analysis of taxonomic profiling results obtained from 2bRAD-M and WMS based on overall divergence, focusing on shared species. Considering only the shared species identified by both methods (represented by retaining only the light blue points and excluding the points on the axes in the image), the resulting similarity

does not improve significantly.

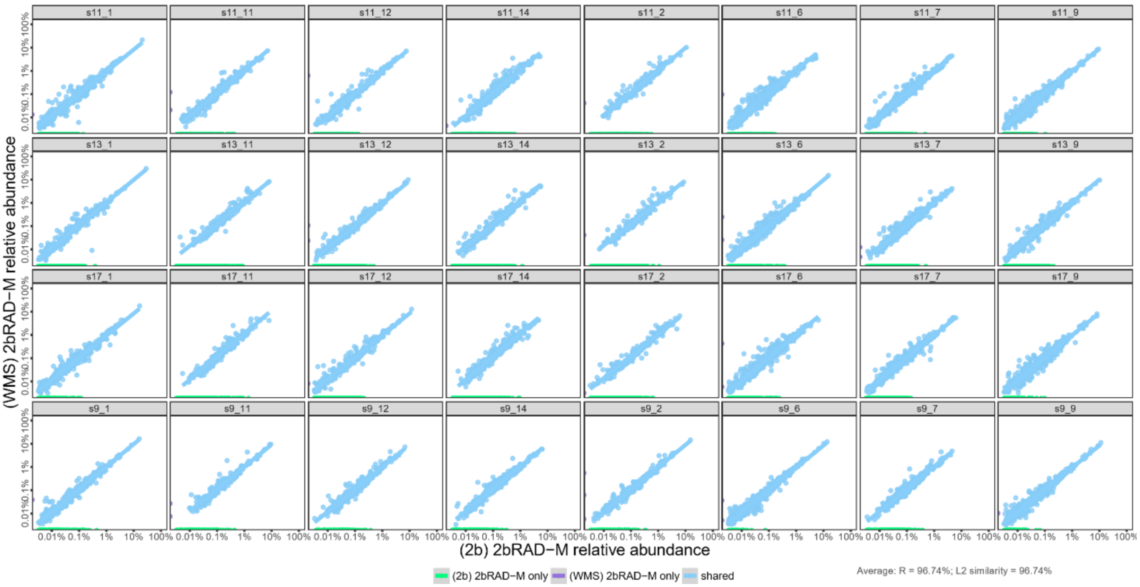

Fig. S6. Comparative analysis of species-level taxonomic profiling results obtained from 2bRAD-M and WMS based on sequencing method disparity. To assess the disparity in sequencing methodologies, we processed both WMS and 2bRAD-M data using the computational pipeline associated with 2bRAD-M and compared the overall similarity of the profiling results. The results showed that the two sequencing methods exhibited extremely high similarity. (WMS) 2bRAD-M: analyzing WMS data using the 2bRAD-M computational pipeline.

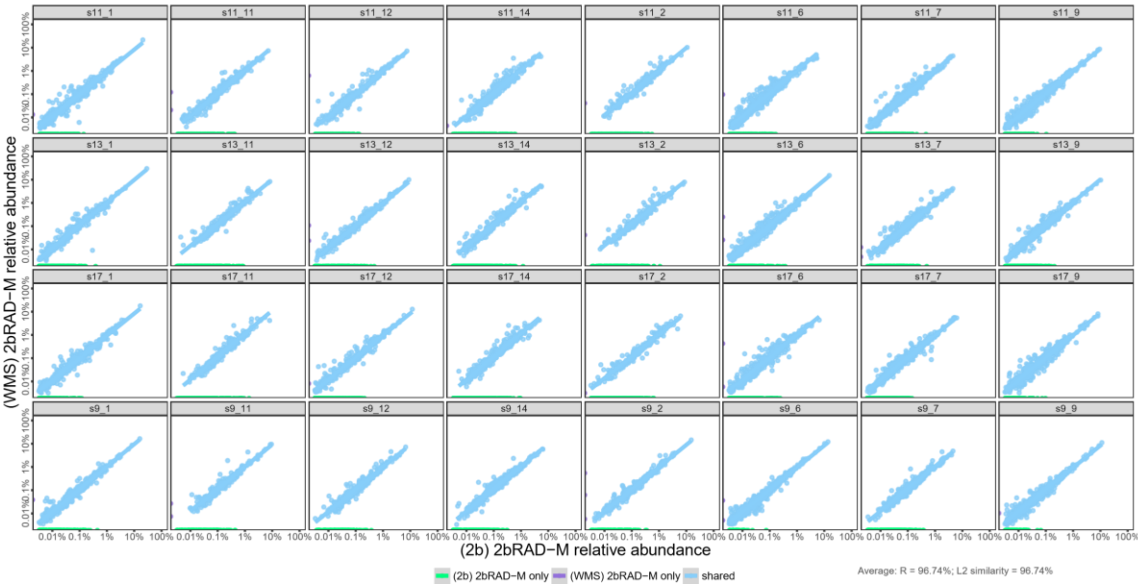

Fig. S7. Comparative analysis of species-level taxonomic profiling results obtained

from 2bRAD-M and WMS based on sequencing method disparity, focusing on shared species. Since the shared species identified using the same 2bRAD-M pipeline accounted for 98.65% of all reads in the 2bRAD-M data and 99.76% in the WMS data, this indicates that the unique species identified in each method involved only a very small portion of the total biomass. Therefore, assessing the similarity between the two sequencing methods using the shared species is reasonable and marginally enhances the similarity indices.

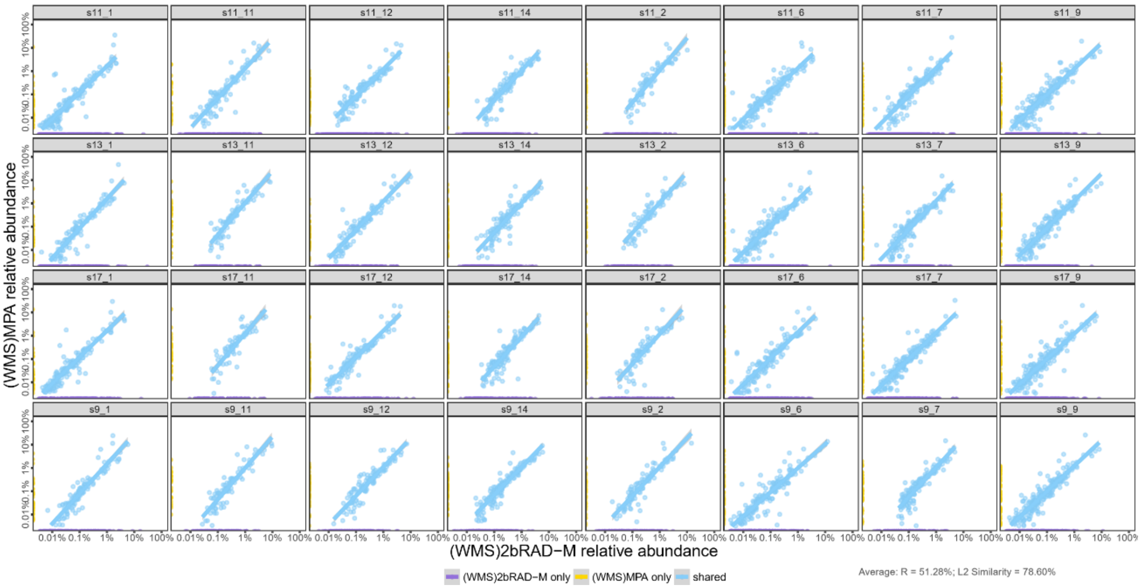

Fig. S8. Comparative analysis of species-level taxonomic profiling results obtained from 2bRAD-M and WMS based on computational pipeline variance. To evaluate the computational pipeline variance, we analyzed the WMS sequencing data using both the MetaPhlAn4 and 2bRAD-M computational pipelines. The resulting similarity was very low, indicating that the heterogeneity in computational analysis methods is the primary reason for the overall differences between WMS and 2bRAD-M.

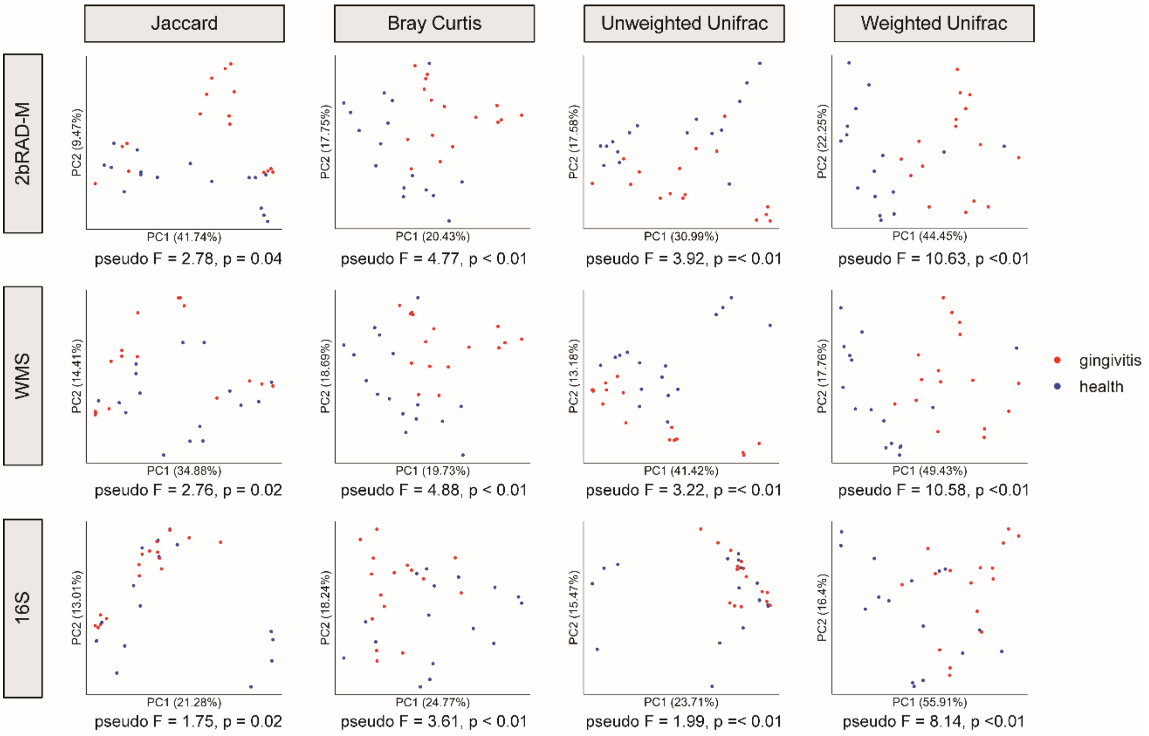

Fig. S9. Beta diversity analysis based on the profiles obtained from 2bRAD-M, WMS, and 16S rRNA sequencing to evaluate their ability in distinguishing between healthy and gingivitis groups. Results obtained from the three sequencing methods all significantly distinguish healthy samples (blue) from samples affected by gingivitis (red). The vertical titles indicate the sequencing methods, while the horizontal titles specify the distance matrix used for the PCoA visualization. The PERMANOVA test was used for significance evaluation. *Pseudo*-F values represent the test statistic, quantitatively indicating the magnitude of the differences.

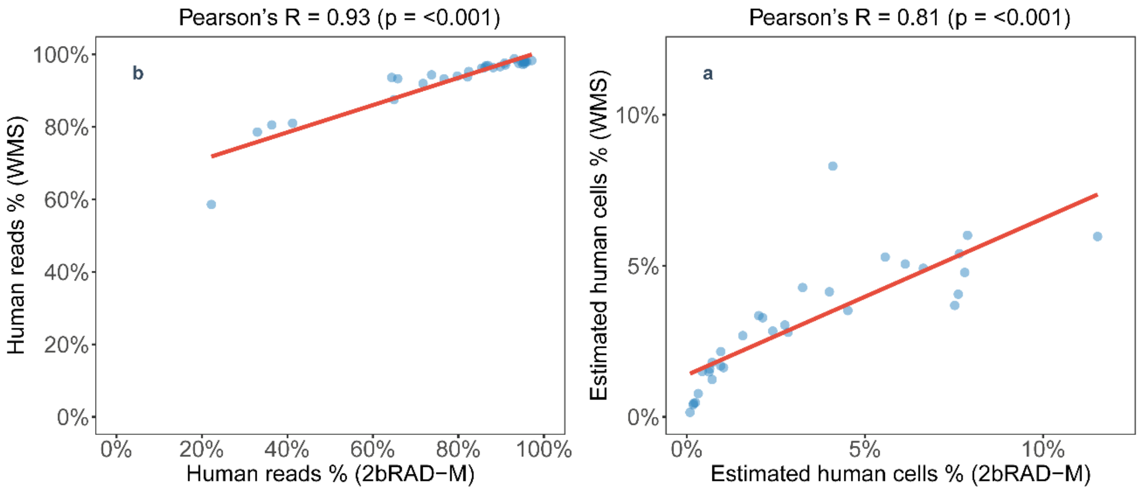

Fig. S10. Scatter plots depict the correlation between (a) human read percentages

and (b) estimated human cell percentages derived from WMS (x-axis) and 2bRAD-M (y-axis).** Each point represents sample-wise measurements, with the red line indicating the linear regression trendline that reflects the overall relationship between methods. Axes are scaled proportionally to the data range: **(a)** 0–100% for human read percentages and **(b)** 0–12% for human cell percentages.

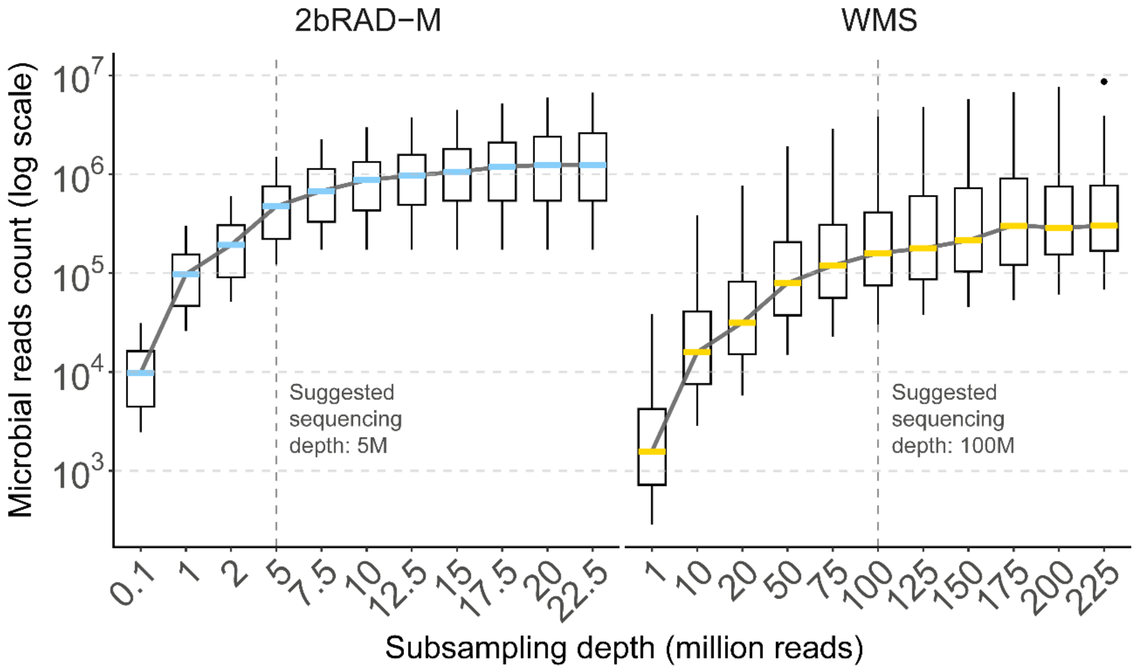

Fig. S11. Microbial read counts or Shannon diversity index measured by 2bRAD-M saturate at a lower sequencing depth than WMS. Log-scale comparison of microbial read recovery between 2bRAD-M (blue line) and whole-metagenome shotgun sequencing (WMS, orange line) across sequencing depths from 0.1 to 225 million reads. Y-axis: microbial read counts (10^3-10^7, log10 scale); X-axis: total sequencing depth (millions of reads). (b) Shannon diversity index measured across subsampling depths. Each colored line represents one sample. For every sequencing depth, three random subsamples were drawn per sample. Saturation of Shannon indices occurs at 5M reads (2bRAD-M) and 100M reads (WMS), capturing over 99% of the maximum recoverable diversity.

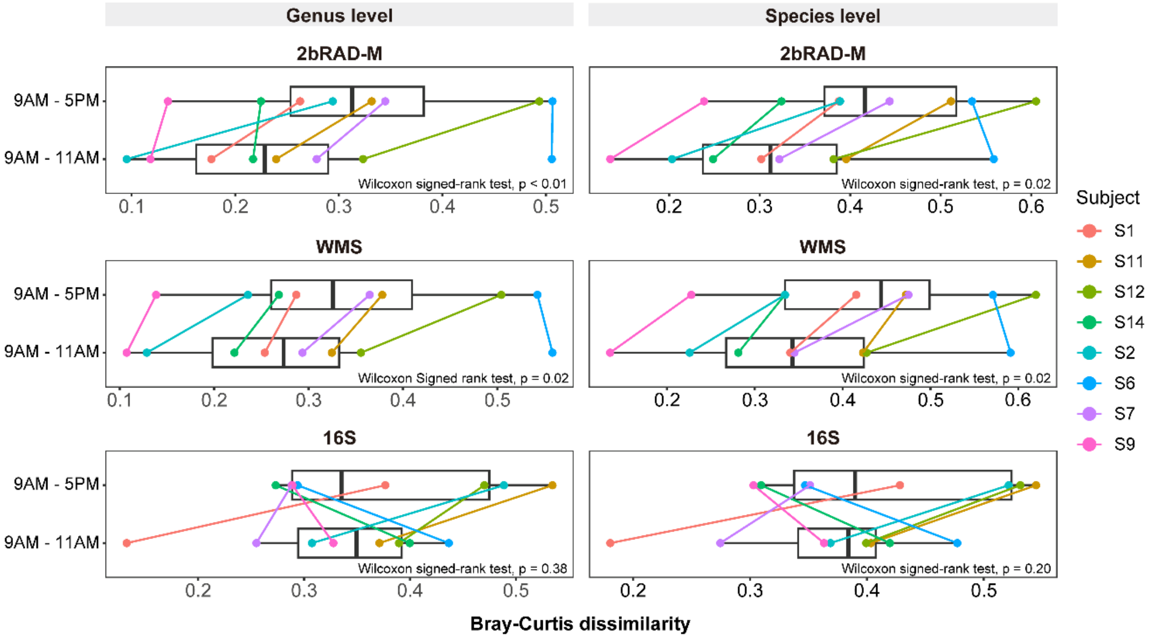

Fig. S12. The diurnal saliva microbiome dynamics measured by genus- and species-level Bray-Curtis dissimilarity. Within-host Bray-Curtis dissimilarity values across time points were calculated from genus-aggregated (left panels) or species-level (right panels) taxonomic profiles generated by 2bRAD-M, WMS, and 16S rRNA gene sequencing. Boxplots depict dissimilarity distributions between time intervals of 9 AM-5 PM and 9 AM-11 AM. Each boxplot represents data from eight hosts. Wilcoxon signed-rank tests revealed that both 2bRAD-M-derived and WMS-derived profiles detected statistically significant differences in genus-level ($p < 0.01$ and $p = 0.02$) and species-level ($p = 0.02$) community structure. At the same time, 16S failed to identify substantial diurnal fluctuations at either taxonomic resolution ($p = 0.38$ and $p = 0.20$).

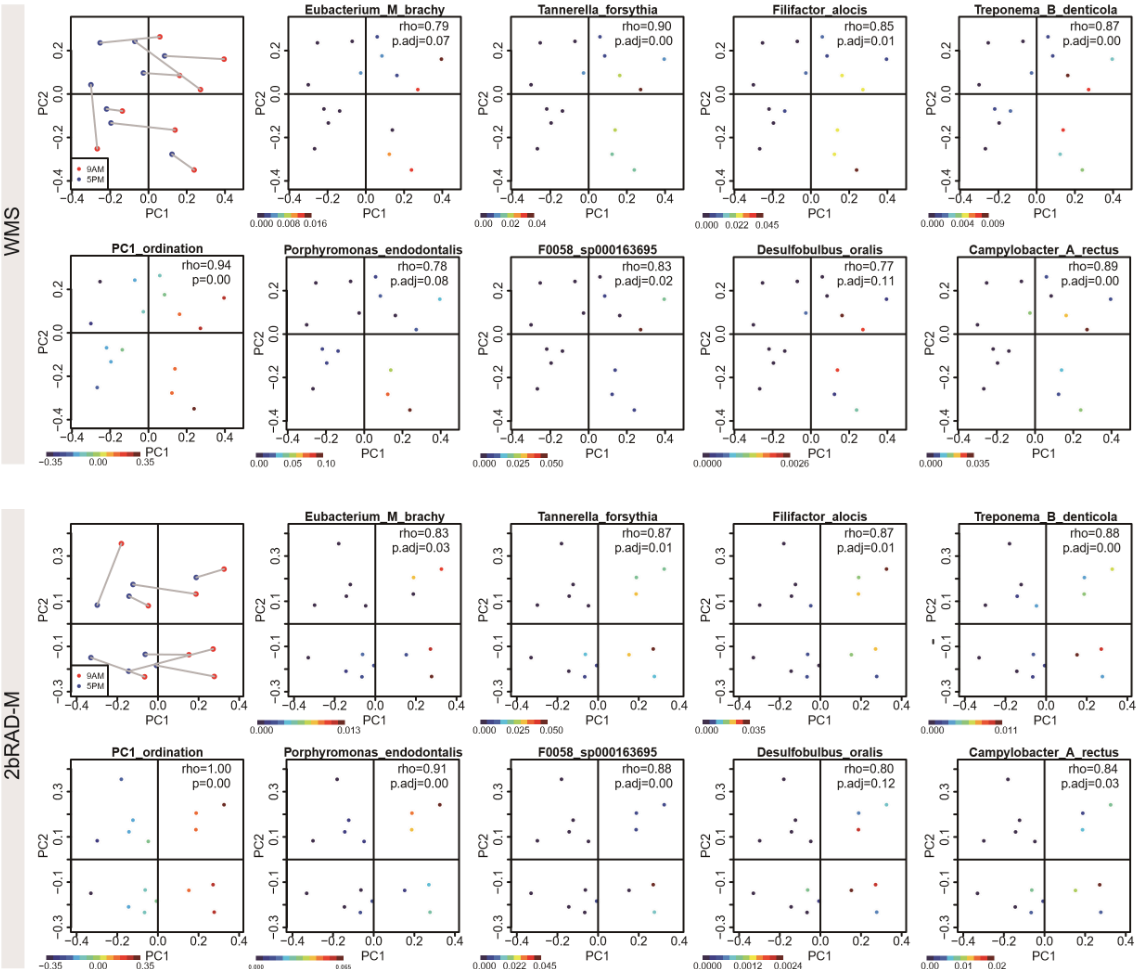

Fig. S13. Decreasing trend in relative abundance of eight shared species from 9 AM to 5 PM across different hosts in 2bRAD-M and WMS analysis. The upper half of the image represents the analysis results from WMS, while the lower half represents the analysis results from 2bRAD-M. Taking the WMS analysis results as an example, the top left image illustrates gradient-like changes in microbial beta diversity between two sampling times, 9 AM (red) and 5 PM (blue), using a PCoA plot of Bray-Curtis dissimilarity matrices. Gray lines connect samples from the same host. The bottom left image displays the ideal gradient for all samples, and the remaining images show the abundance of all eight shared species identified by 2bRAD-M and WMS, with points colored from low abundance (dark blue) to high abundance (brown).

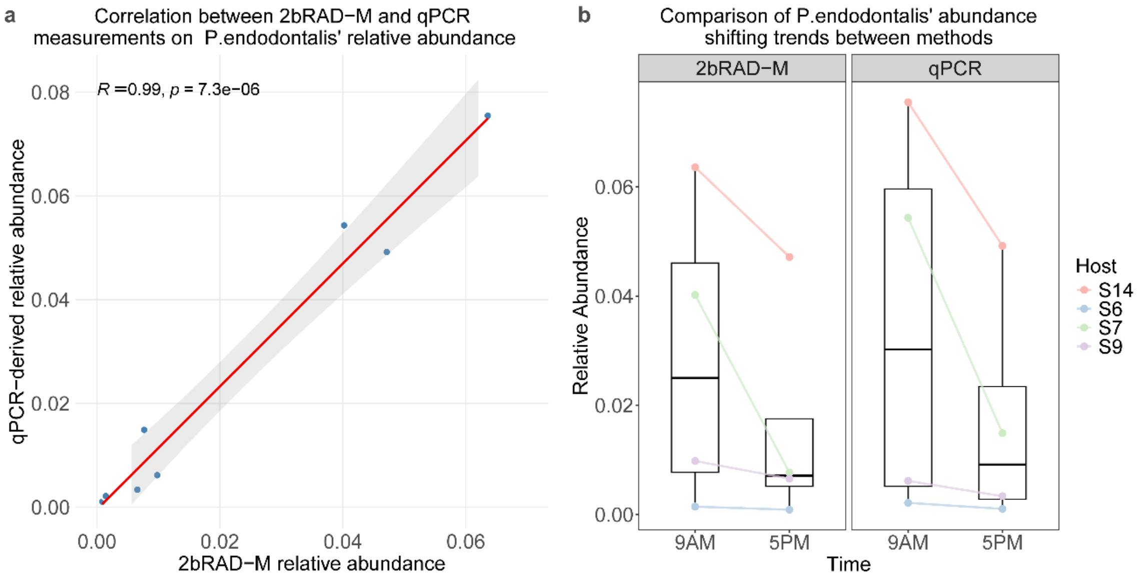

Fig. S14. Comparison of 2bRAD-M and qPCR abundance measurements for *Porphyromonas endodontalis*. (a) Scatter plot comparing *P. endodontalis* relative abundances derived from 2bRAD-M and qPCR assays. For qPCR, the relative abundance of *P. endodontalis* was calculated from the ratio of its absolute abundance to the total bacterial count. Axes: relative abundance values; dashed line: linear regression; report details: Pearson correlation coefficient (R) and p-value. **(b)** Box plots showing diurnal abundance changes (9 AM vs. 5 PM) for *P. endodontalis* measured by 2bRAD-M (left) and qPCR (right). X-axis: time points with host identifiers (S14, S6, S7, S9); Y-axis: relative abundance.

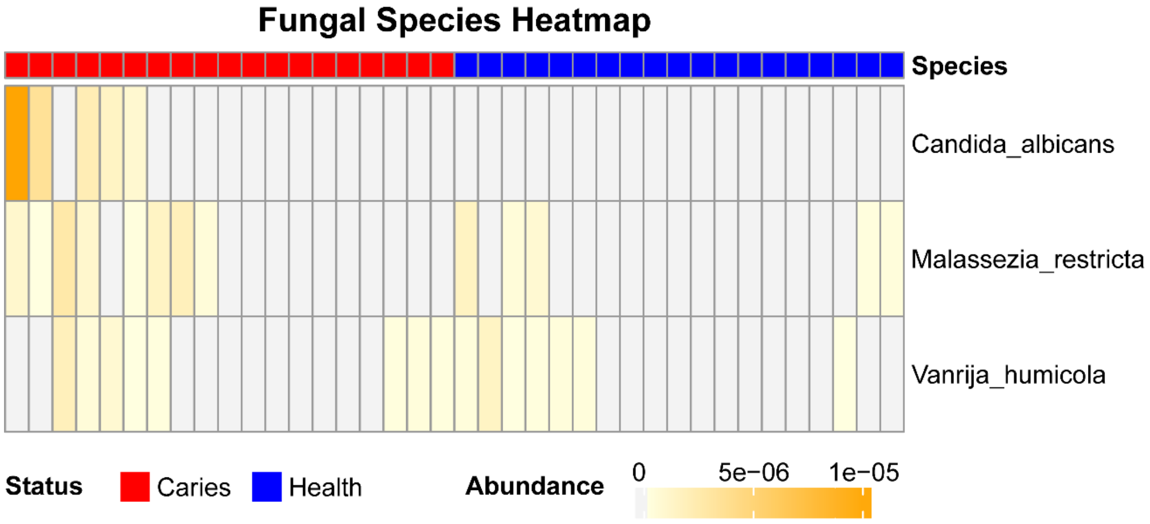

Fig. S15. Fungal species abundance across caries and health states. The heatmap

visualized the relative abundance of three prevalent fungal species between caries and health conditions. Color intensity corresponds to abundance values, which range from 0 to 1×10^{-5}.

Sequencing Method	2bRAD-M				WMS			
Sampleid	Number of classified reads	Number of human reads	Human reads percent	Human cells percent	Number of classified reads	Number of human reads	overall alignment rate	Human cells percent
s11_1	15454920	11844091	76.64%	0.62%	334944805	312302536	93.24%	1.49%
s11_11	7438268	7000865	94.12%	4.00%	223861232	218309473	97.52%	4.14%
s11_12	7142132	6294482	88.13%	2.84%	203705448	196229458	96.33%	2.80%
s11_14	20311069	19227255	94.66%	5.57%	237657603	233070811	98.07%	5.29%
s11_2	8249938	7878980	95.50%	7.65%	177350773	173998843	98.11%	5.40%
s11_6	12618352	9049406	71.72%	0.71%	174227501	160219610	91.96%	1.24%
s11_7	4907508	3617761	73.72%	0.71%	116598570	110010751	94.35%	1.80%
s11_9	12065401	4970301	41.19%	0.24%	209248882	169491594	81.00%	0.47%
s13_1	15449240	13201919	85.45%	1.57%	201598785	193897711	96.18%	2.69%
s13_11	12116810	11539431	95.23%	7.88%	151257465	148701214	98.31%	6.01%
s13_12	8516117	7749097	90.99%	4.52%	234114097	227277965	97.08%	3.52%
s13_14	29905151	27835162	93.08%	4.10%	161541944	159603441	98.80%	8.30%
s13_2	6057326	5885199	97.16%	11.53%	228632799	224746041	98.30%	5.97%
s13_6	12368085	9864140	79.75%	0.95%	266163875	250194043	94.00%	1.69%
s13_7	5007503	4352586	86.92%	2.02%	239377117	232028240	96.93%	3.35%
s13_9	13122818	8449216	64.39%	0.64%	191989939	179740981	93.62%	1.59%
s17_1	16900470	10971044	64.92%	0.32%	191439816	167567271	87.53%	0.77%
s17_11	8394288	8023464	95.58%	6.13%	180051025	176413994	97.98%	5.06%
s17_12	6811134	5613815	82.42%	0.95%	216156388	205932191	95.27%	2.16%
s17_14	27017148	24534010	90.81%	3.25%	129834517	126718489	97.60%	4.28%
s17_2	7374944	7078236	95.98%	6.64%	193892089	189859134	97.92%	4.92%
s17_6	19225356	15791694	82.14%	1.03%	228947767	214707216	93.78%	1.63%
s17_7	4798297	3158108	65.82%	0.43%	191383687	178503565	93.27%	1.50%
s17_9	12949074	4268001	32.96%	0.18%	222988906	175202383	78.57%	0.40%
s9_1	13662310	12259927	89.74%	2.75%	273386757	264118946	96.61%	3.04%
s9_11	7184100	6838640	95.19%	7.80%	227219481	222356984	97.86%	4.78%
s9_12	8108052	7709979	95.09%	7.52%	212695764	206761552	97.21%	3.69%
s9_14	18468188	15911409	86.16%	2.41%	166020016	160010091	96.38%	2.84%
s9_2	8518521	8134472	95.49%	7.62%	213498920	208097397	97.47%	4.06%
s9_6	11279227	2503958	22.20%	0.09%	239541083	140347121	58.59%	0.15%
s9_7	7499388	6482907	86.45%	2.13%	107562210	104184757	96.86%	3.28%
s9_9	12946713	4702665	36.32%	0.19%	244036381	196498094	80.52%	0.45%
Average	11933370	9460694	79.56%	3.28%	205966426	191471934	93.35%	3.09%

Table S1. Summary of human read percentage and estimated human cell proportion in 2bRAD-M and WMS. data.

qPCR plate ID	ID	Sample name	Cp values of triplicate technical replicates for the same sample			Absolute copy numbers derived from Cp values of triplicate replicates (copies/μL DNA)			Mean absolute copy number calculated from triplicate Cp values (copies/μL DNA)	Dilution Factor	Absolute copy number in undiluted DNA sample (copies/μL DNA)
			Cp1	Cp2	Cp3	Copies1	Copies2	Copies3	Mean		
P1	1	S17_14	17.75	17.71	17.58	5.50E+04	5.65E+04	6.16E+04	5.77E+04	1	5.77E+04
P1	2	S17_6	27.98	28.19	27.97	6.43E+01	5.60E+01	6.48E+01	6.17E+01	3	1.85E+02
P1	3	S17_7	15.57	14.89	14.70	2.32E+05	3.63E+05	4.12E+05	3.36E+05	1	3.36E+05
P1	4	S17_9	16.82	16.63	16.48	1.02E+05	1.15E+05	1.27E+05	1.15E+05	1	1.15E+05
P1	5	S9_14	17.13	16.99	16.96	8.29E+04	9.09E+04	9.27E+04	8.88E+04	1	8.88E+04
P1	6	S9_6	20.76	20.74	20.52	7.55E+03	7.65E+03	8.84E+03	8.01E+03	1	8.01E+03
P1	7	S9_7	16.01	15.75	14.88	1.74E+05	2.06E+05	3.66E+05	2.48E+05	3	7.45E+05
P1	8	S9_9	17.34	16.68	16.72	7.21E+04	1.12E+05	1.09E+05	9.74E+04	1	9.74E+04
P1	9	NTC	36.62	Undet	34.46	2.15E-01	Undet	8.94E-01	/	/	/

Standard Curve	ID	Standard curve concentration gradient (copies/μL)	log10 values of standard curve concentrations	Cp values of triplicate technical replicates for the same sample			Mean Cp value of triplicates	Standard Curve	
				Cp1	Cp2	Cp3		X axis	Y axis
P.e Standard Curve	1	2.92E+06	6.465	11.79	11.50	11.73	11.673	6.465	11.673
	2	2.92E+05	5.465	15.35	15.25	15.31	15.303	5.465	15.303
	3	2.92E+04	4.465	18.77	18.57	18.45	18.597	4.465	18.597
	4	2.92E+03	3.465	22.59	22.15	21.99	22.243	3.465	22.243
	5	2.92E+02	2.465	25.79	26.08	25.74	25.870	2.465	25.870
	6	2.92E+01	1.465	29.00	29.18	28.91	29.030	1.465	29.030

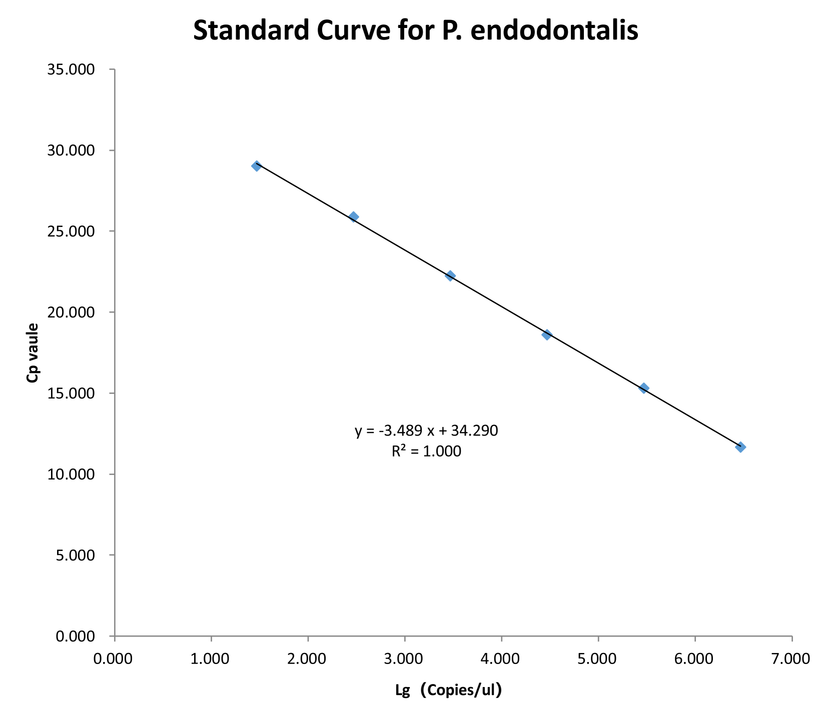

qPCR plate ID	ID	Sample name	Cp values of triplicate technical replicates for the same sample			Absolute copy numbers derived from Cp values of triplicate replicates (copies/μL DNA)			Mean absolute copy number calculated from triplicate Cp values (copies/μL DNA)	Dilution Factor	Absolute copy number in undiluted DNA sample (copies/μL DNA)
			Cp1	Cp2	Cp3	Copies1	Copies2	Copies3	Mean		
P1	1	S17_14	16.77	17.00	16.93	1.27E+05	1.10E+05	1.15E+05	1.17E+05	10	1.17E+06
P1	2	S17_6	20.52	20.49	20.42	1.15E+04	1.17E+04	1.23E+04	1.18E+04	15	1.77E+05
P1	3	S17_7	12.42	12.21	12.24	2.07E+06	2.37E+06	2.32E+06	2.25E+06	10	2.25E+07
P1	4	S17_9	11.59	11.66	11.68	3.52E+06	3.37E+06	3.32E+06	3.40E+06	10	3.40E+07
P1	5	S9_14	17.06	16.82	16.81	1.06E+05	1.23E+05	1.24E+05	1.18E+05	10	1.18E+06
P1	6	S9_6	15.20	15.20	14.89	3.48E+05	3.48E+05	4.25E+05	3.74E+05	10	3.74E+06
P1	7	S9_7	13.83	13.62	13.64	8.38E+05	9.59E+05	9.46E+05	9.14E+05	15	1.37E+07
P1	8	S9_9	12.94	12.87	12.72	1.48E+06	1.55E+06	1.71E+06	1.58E+06	10	1.58E+07
P1	9	NTC	Undet	Undet	Undet	Undet	Undet	Undet	/	/	/

Standard Curve	ID	Standard curve concentration gradient (copies/μL)	log10 values of standard curve concentrations	Cp values of triplicate technical replicates for the same sample			Mean Cp value of triplicates	Standard Curve	
				Cp1	Cp2	Cp3		X axis	Y axis
16SV3V4Standard Curve	1	1.62E+07	7.210	9.47	9.07	8.98	9.173	7.210	9.173
	2	1.62E+06	6.210	12.85	12.92	12.65	12.807	6.210	12.807
	3	1.62E+05	5.210	16.64	16.53	16.50	16.557	5.210	16.557
	4	1.62E+04	4.210	19.72	19.95	19.95	19.873	4.210	19.873
	5	1.62E+03	3.210	23.23	23.54	23.64	23.470	3.210	23.470
	6	1.62E+02	2.210	27.32	26.97	27.48	27.257	2.210	27.257

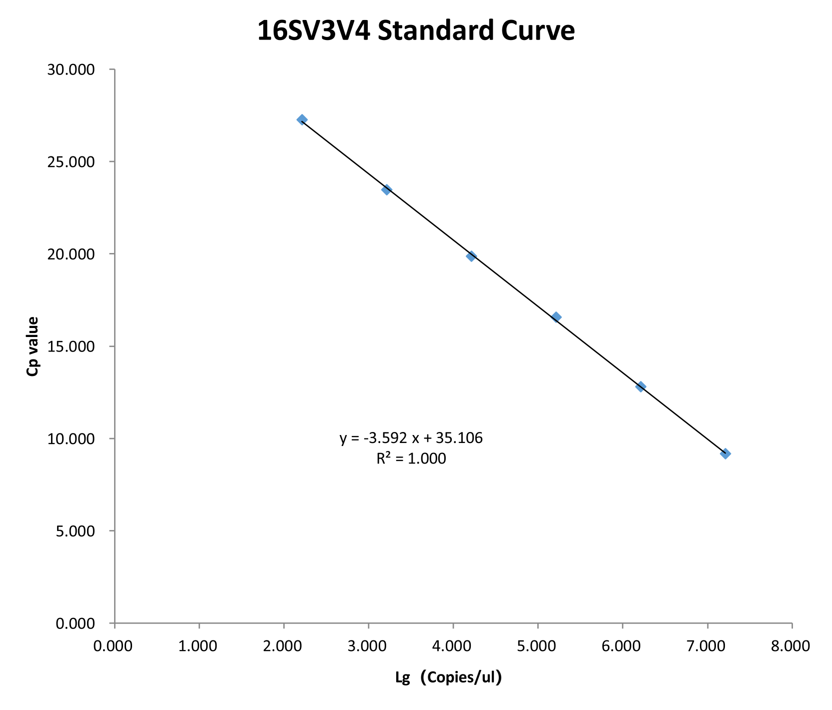

Table S2. qPCR abundance measurements for *Porphyromonas endodontalis* and total bacterial load.
